# Impact of *Paraburkholderia phytofirmans* PsJN on Grapevine Phenolic Metabolism

**DOI:** 10.3390/ijms20225775

**Published:** 2019-11-16

**Authors:** Lidiane Miotto-Vilanova, Barbara Courteaux, Rosa Padilla, Fanja Rabenoelina, Cédric Jacquard, Christophe Clément, Gilles Comte, Céline Lavire, Essaïd Ait Barka, Isabelle Kerzaon, Lisa Sanchez

**Affiliations:** 1 Unité de Recherche EA 4707 Résistance Induite et Bioprotection des Plantes (RIBP), Université de Reims Champagne-Ardenne, SFR Condorcet FR CNRS 3417, 51687 Reims Cedex 2, France; lidianemiotto@gmail.com (L.M.-V.); barbara.courteaux@univ-reims.fr (B.C.); clarisse.rabenoelina@univ-reims.fr (F.R.); cedric.jacquard@univ-reims.fr (C.J.); christophe.clement@univ-reims.fr (C.C.); ea.barka@univ-reims.fr (E.A.B.); 2 Ecologie Microbienne, Université Lyon 1, CNRS, INRA, UMR 5557, 69622 Villeurbanne, France; ropagui@hotmail.com (R.P.); gilles.comte@univ-lyon1.fr (G.C.); celine.lavire@univ-lyon1.fr (C.L.); isabelle.kerzaon@univ-lyon1.fr (I.K.)

**Keywords:** *Vitis vinifera*, beneficial bacterium, phenolic compounds, qRT-PCR, UHPLC-UV/DAD-MS QTOF

## Abstract

Phenolic compounds are implied in plant-microorganisms interaction and may be induced in response to plant growth-promoting rhizobacteria (PGPRs). Among PGPR, the beneficial bacterium *Paraburkholderia phytofirmans* PsJN was previously described to stimulate the growth of plants and to induce a better adaptation to both abiotic and biotic stresses. This study aimed to investigate the impact of PsJN on grapevine secondary metabolism. For this purpose, gene expression (qRT-PCR) and profiling of plant secondary metabolites (UHPLC-UV/DAD-MS QTOF) from both grapevine root and leaves were compared between non-bacterized and PsJN-bacterized grapevine plantlets. Our results showed that PsJN induced locally (roots) and systemically (leaves) an overexpression of *PAL* and *STS* and specifically in leaves the overexpression of all the genes implied in phenylpropanoid and flavonoid pathways. Moreover, the metabolomic approach revealed that relative amounts of 32 and 17 compounds in roots and leaves, respectively, were significantly modified by PsJN. Once identified to be accumulated in response to PsJN by the metabolomic approach, antifungal properties of purified molecules were validated in vitro for their antifungal effect on *Botrytis* *cinerea* spore germination. Taking together, our findings on the impact of PsJN on phenolic metabolism allowed us to identify a supplementary biocontrol mechanism developed by this PGPR to induce plant resistance against pathogens.

## 1. Introduction

Phenolic compounds (also named polyphenols) are widely distributed in the plant kingdom with more than 8000 known phenolic structures [1,2]. This class of secondary metabolites naturally comprises a large chemical diversity, ranging from simple phenolic acids to complex polymerized tannins. While some are the plant structural constituents, such as hydroxyl cyclic acids, others, such as proanthocyanidins, are induced in response to stress [3,4,5]. In plants, they are mainly described as involved in resistance against pathogens and herbivores, limitation of radiation damage [6,7], or act as signaling molecules in plant microbes symbioses [8].

Grapevine is one of the most cultivated plant species worldwide and is particularly rich in secondary metabolites, including phenolic compounds. The metabolism and content of phenolic compounds in grapevine can be modified during biotic and abiotic stress [4,9]. A study [10] demonstrated that total phenol content and antioxidant activity were higher in leaves of cold-tolerant cultivar than in susceptible ones. In grapevine plants affected by esca (a destructive disease which affects the vines), higher amounts of phenolic compounds have been reported in different tissues. In particular, resveratrol and ε-viniferin (among other stilbene polyphenols) accumulated in the symptomatic wood of plants infected with esca [11]. To date, most of the studies analyzing the effect of microorganisms on grapevine phenolic metabolism have concerned the impact of pathogens.

Elicitation of plant secondary metabolites by beneficial microorganisms or molecules has been previously used [12,13,14,15,16,17] to improve resistance against biotic and abiotic stresses. The ability of PGPRs (plant growth-promoting rhizobacteria) to increase the crop productivity either by stimulating plant growth or by protecting them against abiotic or biotic stresses has been widely demonstrated in many plant species [18]. Interestingly, plant secondary metabolites, including phenolic compounds, may be induced in response to colonization by PGPRs. Indeed, if inoculation with PGPRs can reproducibly alter the secondary metabolism, their application can provide a nutritional value added to a crop and contribute to the nutritional security [19]. In this context, seed treatment with two *Pseudomonas* PGPR strains induced phenolic acid synthesis and accumulation of total phenols at different growth stages of chickpea plants [13]. Similarly, inoculation of tomato seeds with *Bacillus* PGPR strains increased the total phenol content in plants [20]. The reduction rate of the bacterial canker disease in tomatoes was reported to be directly proportional to the increase in total phenols, showing the involvement of this type of compounds in the plant defense. Biotic elicitation with PGPR appears as a useful strategy to both improve biomass production and to trigger secondary metabolism [21,22].

Among PGPR, the beneficial endophyte bacterium *Paraburkholderia phytofirmans* PsJN has been described to stimulate the growth of bacterized plants and to induce a better adaptation to both abiotic [15,23,24] and biotic stresses [25]. This strain is also known to modulate primary and secondary metabolism in different plants [24,26,27,28,29]. In grapevine, [15] reported that PsJN improved the growth of the plantlets and increased the total phenol content in leaves. The present study aimed to investigate the effect of PsJN root-inoculation on grapevine secondary metabolism. First, we analyzed the colonization profile of PsJN in grapevine plantlets and observed the impact of this beneficial bacterium on the plant phenotype. Moreover, to gain more insights into the secondary metabolism modifications in response to PsJN, analyses of gene expression and profiling of plant secondary metabolites from both grapevine roots and leaves were compared between control and PsJN-bacterized plants. Finally, we tested the direct antimicrobial effect of the pure molecules identified by our metabolomic approach on *Botrytis* spore germination in vitro.

## 2. Results

### 2.1. Phenotypic Changes Induced by PsJN

Endophytic and rhizospheric populations of PsJN, as well as the migration of the bacteria to the leaves, were followed at 1, 4, and 7 days after root-inoculation (Supplemental Figure S1). The results showed that the rhizospheric population detected 2 h after inoculation was 4.4 log CFU. g^−1^ fresh weight (FW), which corresponds to the initial inoculum. The bacterium PsJN proliferated at the rhizosphere level to reach a maximum density of 7.8 log CFU. g^−1^ FW 7 days later. Endophytic root colonization was detected from the first day after bacterization, reaching a maximum density of 5.5 log CFU. g^−1^ FW 7 days after inoculation. At the foliar level, the presence of the bacterium was observed only at the 7th day after bacterization with a slight density of 1.8 log CFU. g^−1^ FW.

**Figure 1:**
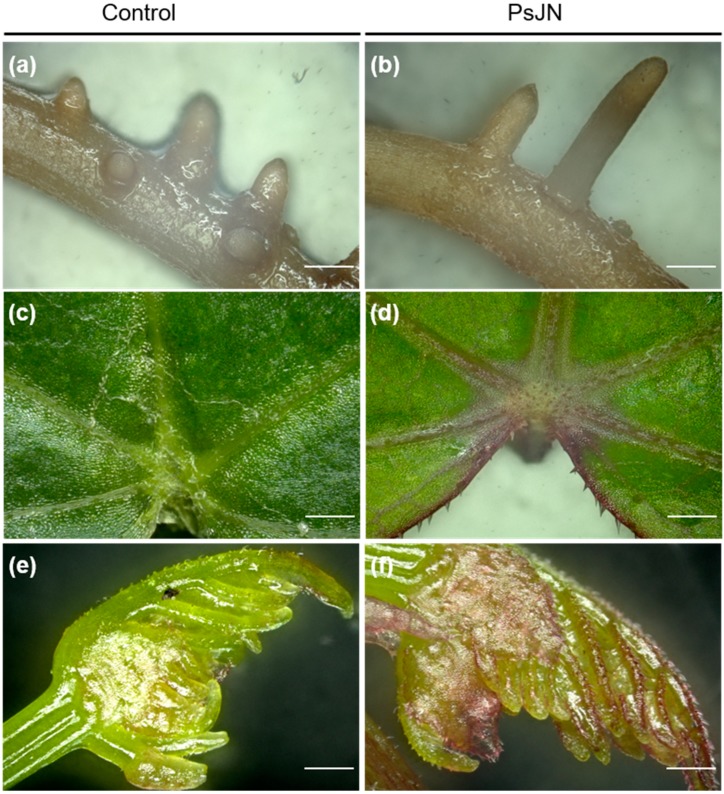
Microscopic observations of grapevine plantlets roots (**a**,**b**) and leaves (**c**–**f**), root-inoculated (on the right) or not (on the left) by *P. phytofirmans* strain PsJN. Four weeks-old plantlets were inoculated with 200 µL of bacterial inoculum (10^9^ CFU. mL^−1^) or PBS 10 mM (control). Observations were realized 7 days after inoculation using a 3D microscope. Scale bars = 500 μm. CFU, colony-forming unit.

In order to determine the impact of *P. phytofirmans* PsJN on grapevine plantlets, 3D microscopic observations of the roots and leaves were carried out 7 days after root-inoculation. The results showed that the colonization by the PsJN strain did not produce visible lesions on the studied organs (Figure 1). No difference was observed between non-inoculated and inoculated roots (Figure 1a,b). However, plants inoculated with the bacteria presented reddish leaves compared to control ones (Figure 1c–f).

**Figure 2:**
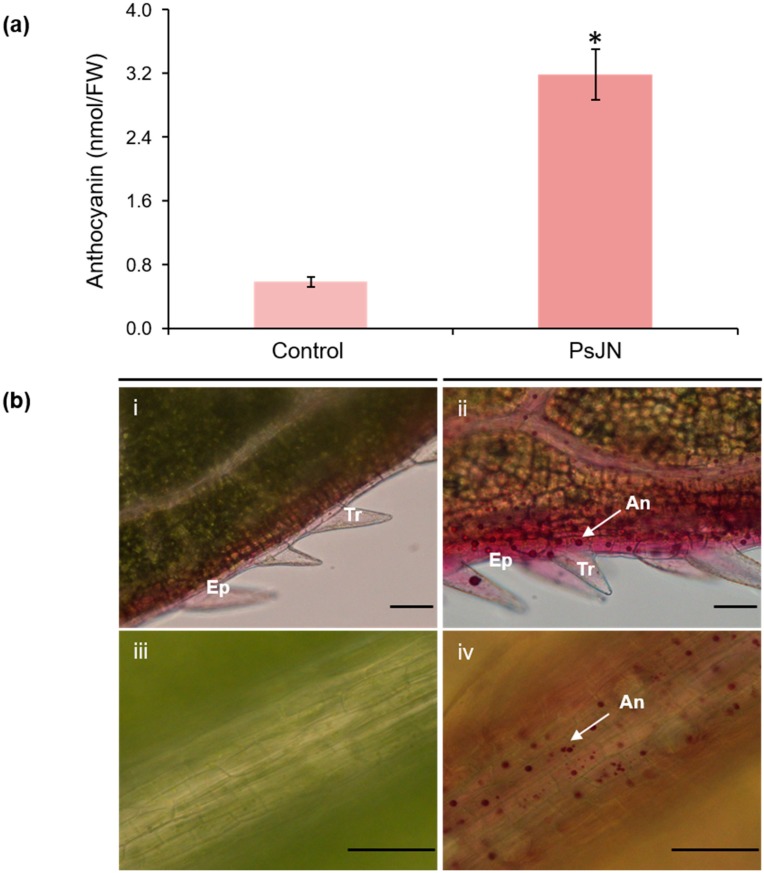
Anthocyanin accumulation in leaves 7 days after root-inoculation by *P. phytofirmans* PsJN or PBS (control). (**a**) Quantification of anthocyanin total content (*n* = 8) and (**b**) microscopic observations (i,ii) leaf border; (iii,iv) leaf vein. An: anthocyans; Ep: epiderm; Tr: trichome. Scale bars = 100 μm. * indicates significant differences (*p* ≤ 0.05) as determined by Tukey test analysis.

As anthocyanins may be responsible for the red color of leaves, we monitored their total content, which was higher in bacterized-plantlets compared to control ones (Figure 2a). Moreover, 3D microscopic observations revealed that the presence of anthocyanins was greater in the epidermis of the leaf and the main vein in bacterized plants (Figure 2b).

### 2.2. Modulation of Phenolics-Related Gene Expression in Roots and Leaves

**Figure 3:**
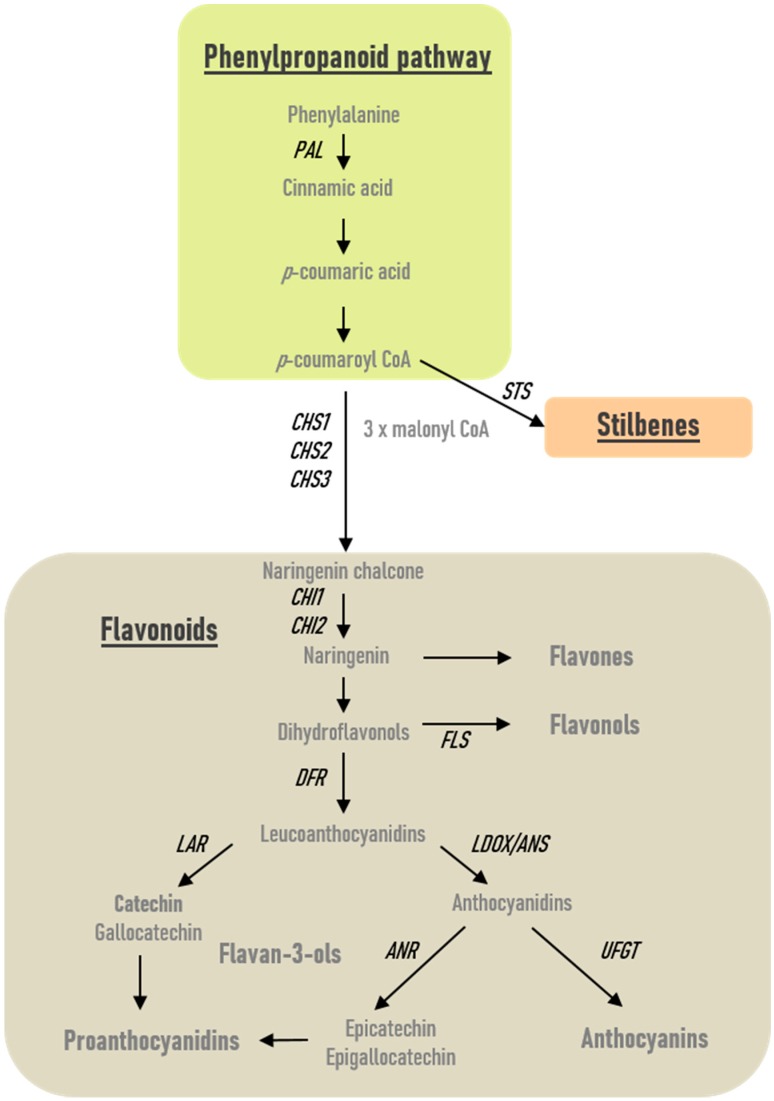
General phenylpropanoid and flavonoid biosynthesis pathways in plants. Phenylalanine ammonia-lyase (*PAL*), which catalyzes the first step of the phenylpropanoid pathway, a stilbene synthase (*STS*), three chalcone synthase (*CHS1*, *CHS2*, *CHS3*), two chalcone isomerase 1 and 2 (*CHI1*, *CHI2*), a flavonol synthase (*FLS*), one dihydroflavonol reductase (*DFR*), two leucoanthocyanidin reductase (*LAR1*, *LAR2*), an anthocyanidin reductase (*ANR*), a leucoanthocyanidin dioxygenase (*LDOX*), and an UDP-glucose: flavonoid 3-*O*-glucosyl transferase (*UFGT*) were studied in the present work and localized here in the general biosynthesis pathway.

Anthocyanins are the final products of a specific branch of the flavonoid pathway (Figure 3). In order to determine changes in transcript accumulation induced by PsJN, we monitored by qRT-PCR the expression of genes encoding for phenylalanine ammonia-lyase (*PAL*), which catalyzes the first step of the phenylpropanoid pathway, a stilbene synthase (*STS*), three chalcone synthase (*CHS1*, *CHS2*, *CHS3*), two chalcone isomerase 1 and 2 (*CHI1*, *CHI2*), a flavonol synthase (*FLS*), one dihydroflavonol reductase (*DFR*), two leucoanthocyanidin reductase (*LAR1*, *LAR2*), an anthocyanidin reductase (*ANR*), a leucoanthocyanidin dioxygenase (*LDOX*), and an UDP-glucose: flavonoid 3-*O*-glucosyl transferase (*UFGT*) in roots and leaves of uninoculated and PsJN-inoculated plantlets. A kinetic was realized at 1, 4, and 7 days after bacterization.

#### 2.2.1. Roots

**Figure 4:**
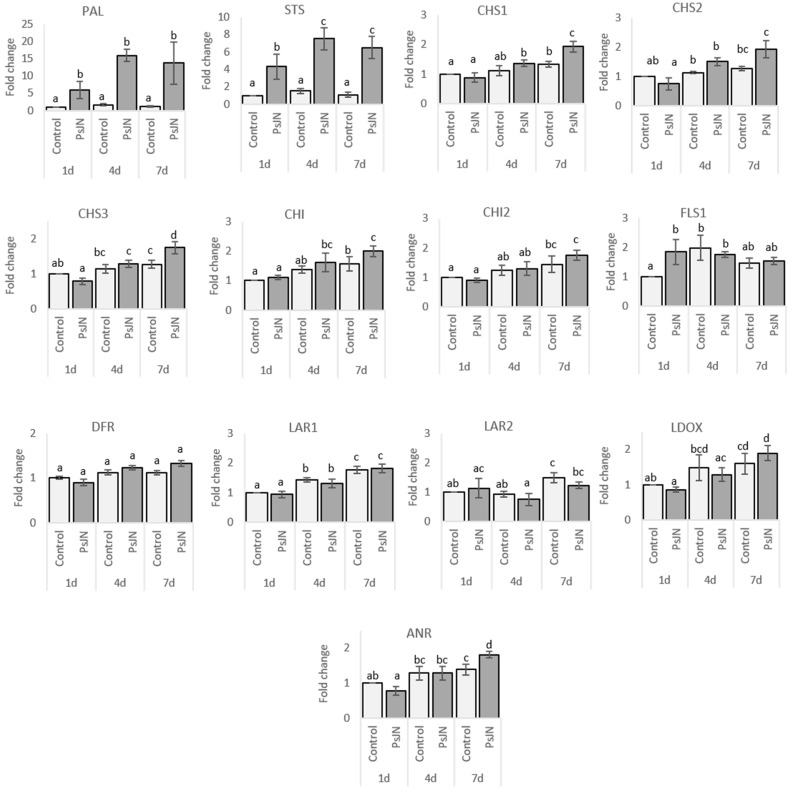
Gene expression in roots of grapevine inoculated or not with *P. phytofirmans* PsJN. Transcript accumulation was determined by qRT-PCR one, four, and seven days after inoculation by PsJN. Gene transcript levels were normalized using two reference genes (*EF1*α, *60* *RSP*) as internal controls. Results are expressed as the fold increase in transcript level compared to control, treated with buffer. Values shown are means +/− SD of two independent repetitions; each repetition was realized in duplicates. Letters a–d indicate significant differences (*p* ≤ 0.05) between treatments, as determined by Tukey analysis.

In roots, the transcript levels of *PAL* and *STS* were strongly up-regulated from one day and throughout the kinetics at 4 and 7 days after bacterization with PsJN (Figure 4). *CHS* is encoded by three genes in grapevine [30]. In response to PsJN, we observed only a slight activation of *CHS1*, *CHS2*, *CHS3*, *CHI1*, and *ANR* at 7 days after bacterization. For *FLS1*, a significant but slight induction was observed in response to PsJN one day after bacterization, although this effect disappeared at 4 and 7 days. No amplification was obtained in roots using primers targeting the *UFGT* gene, suggesting a too lower expression to be detected.

#### 2.2.2. Leaves

**Figure 5:**
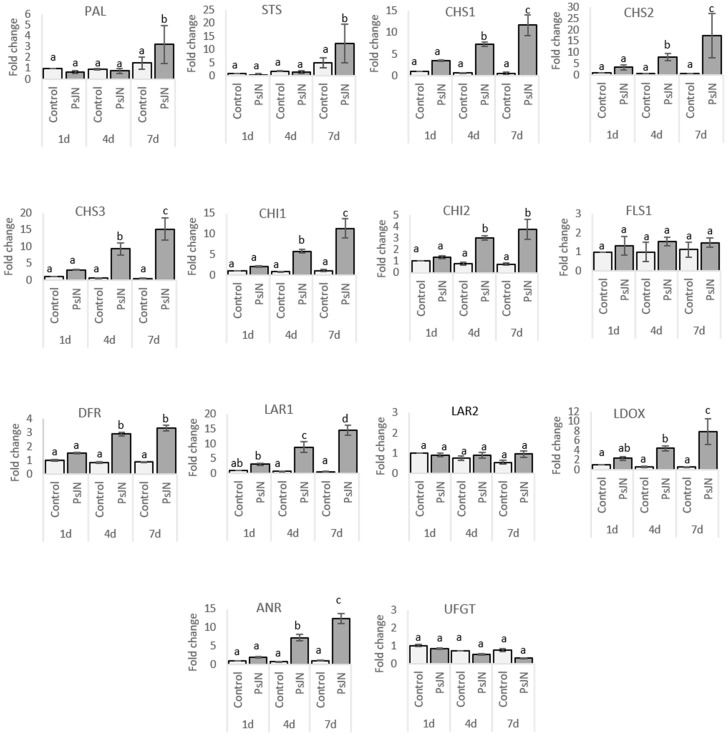
Gene expression in leaves of grapevine inoculated or not with *P. phytofirmans* PsJN. Transcript accumulation was determined by qRT-PCR one, four, and seven days after inoculation by PsJN. Gene transcript levels were normalized using two reference genes (*EF1*α, *60* *RSP*) as internal controls. Results are expressed as the fold increase in transcript level compared to control, treated with buffer. Values shown are means +/− SD of two independent repetitions; each repetition was realized in duplicates. Letters a–d indicate significant differences (*p* ≤ 0.05) between treatments, as determined by Tukey analysis.

In leaves, no significant difference was observed in transcript accumulation for *FLS1 LAR2* and *UFGT* in response to PsJN (Figure 5). However, the expression of *CHS1*, *CHS2*, *CHS3*, *CHI*, *CHI2*, *LAR1*, *ANR,* and *LDOX* was up-regulated in bacterized plantlets four and seven days after bacterization. For *PAL* and *STS*, a significant overexpression in response to PsJN was observed only seven days after bacterization.

### 2.3. Phenolics Profiles in Response to P. phytofirmans PsJN

**Figure 6:**
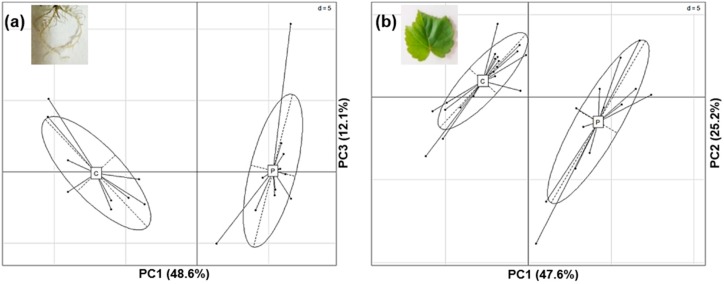
Comparison of root and leave phenolic profiles between control and bacterized plantlets. Principal component analysis (PCA) performed on chromatographic data obtained for each methanolic extract of grapevine after inoculation or not with PsJN. Analyses were based on peak areas and retention times. This figure represents both the data obtained in the first and second biological repetitions. Each point represents the extract of pooled samples of the same treatment (nine plants/pool). (**a**) Root extracts (C = control plants, n_1_ = 5 + n_2_ = 4; P = PsJN inoculated-plants, n_1_ = 8 + n_2_ = 4; data matrix of 85 peaks); (**b**) Leave extracts (C = control plants, n_1_ = 9 + n_2_ = 11; P = PsJN inoculated-plants, n_1_ = 9 + n_2_ = 4; data matrix of 32 peaks). n_1_: number of replicates in the first biological repetition; n_2_: number of replicates in the second biological repetition.

In order to deeper investigate the quantitative and qualitative modifications of phenolic compounds of grapevine plantlets inoculated with *P. phytofirmans* PsJN, metabolic changes in roots and leaves in response to PsJN were evaluated by comparison with control plantlets. The extracts obtained from roots and leaves of vitroplantlets were analyzed by UHPLC-UV/DAD-MS QTOF and showed a more complex composition of the obtained extracts for roots than leaves. Indeed, the analysis data processing has led to integrating 85 and 32 peaks for roots and leaves, respectively, on the chromatogram at 280 nm (Supplemental Figure S2a,b). The retention times and intensity of signals were reproducible and stable along the analytical process, indicating the reliability of metabolomic investigation. Data matrixes obtained from chromatographic profiles were further compared by principal component analysis (PCA), showing clear discrimination between control and bacterized plantlets, both for roots and leaves (Figure 6). The control and bacterized samples were mainly separated by the PC1, which represented 48.6% and 47.6% of the variation among the samples, respectively, in roots and leaves (Figure 6a,b). This figure represents both the data obtained in the first and second biological repetitions. The two independent biological repetitions of the experiment showed similar chromatographic profiles and discriminations between the two conditions for both roots and leaves, strengthening these results.

A PLS-DA (partial least squares discriminant analysis) was also performed on the data, allowing to highlight the most discriminant metabolites in each dataset (variable importance for the projection, VIP score >1). The statistical test revealed that relative amounts of 32 and 17 compounds in roots and leaves, respectively, were significantly modified by the beneficial bacterium. A smaller number of metabolites were modified by PsJN in leaves compared to the roots. However, discriminant compounds highlighted in the leaves represented 53% of the detected metabolites against around 38% for the roots, as the composition detected for the obtained both extracts was initially different.

#### 2.3.1. Compounds Identification

The UHPLC-UV/DAD-MS QTOF data were explored in order to identify the discriminant compounds brought out by statistical analyses. Study of the spectral data (UV; MS and MS/MS in negative and positive ionization mode) allowed annotating or identifying some molecules in different classes of secondary metabolites by comparison to bibliographical data or analyses of some standard compounds available in the lab. The chemical data of the annotated compounds are shown in Table 1 and Table 2 for roots and leaves, respectively.

**Organic acid.** At the beginning of the root extracts analysis, a very polar compound was detected as a discriminant metabolite. This peak **[R4]** observed at 0.96 min as an ionic species [M-H]^−^ at *m*/*z* 191.0191 was assigned as citric acid by the comparison of its retention time and mass spectral data to the authentic standard and in accordance with the literature [31,32].

**Hydroxycinnamic esters.** In leaves extracts analyses, two discriminant peaks **[L5; L9]** showed UV-vis spectra characteristic of hydroxycinnamic acid (HCA) derivatives, with major λ_max_ at 322 nm (298 sh) and 312 nm (295 sh), respectively (Table 2). They were observed as precursor ions [M-H]^−^ at *m*/*z* 311.0411 and at *m*/*z* 295.0457, and both showed product ions due to the loss of 132 uma (corresponding to the loss of a tartaric acid moiety) observed at *m*/*z* 179.0332 (which could be caffeic acid) and at *m*/*z* 163.0386 (coumaric acid), respectively. Other product-ions were observed at *m*/*z* 149.0085 (tartaric acid), produced due to the loss of the HCA moiety (caffeic or coumaric acid), and at *m*/*z* 135.0439 or *m*/*z* 119.0498, respectively, derived from the decarboxylation of the HCA moiety. As these data were in accordance with fragmentation patterns described in the literature, these compounds **[L5; L9]** were assigned to caffeoyl-tartaric (caftaric) and coumaroyl-tartaric (coutaric) acids, respectively, without isomer type determination [33,34,35]. Two other hydroxycinnamoyl tartaric acids were detected in the leaves extracts (compounds **[L6; L13]** as a coutaric and fertaric acid isomers, respectively) but were not discriminant compounds.

**Phenolic acid derivatives.** Three glycosylated phenolic acids were identified as discriminant compounds in roots extracts **[R12; R18; R26]**. They were detected in negative ionization mode as precursor ions [M-H]^−^ at *m*/*z* 315.0718 **[R12]**, *m*/*z* 299.0769 **[R18]**, and *m*/*z* 329.0867 **[R26]**, and they all showed in MS^2^ a neutral loss of 162 uma (loss of hexose), leading to the phenolic acid aglycon product ions at *m*/*z* 153 (protocatechuic acid), *m*/*z* 137 (hydroxybenzoic acid), and *m*/*z* 167 (vanillic acid), respectively. Among the product ions observed, MS^2^ spectra of **[R12]** and **[R26]** also presented the common fragment ions [M-H-CO_2_]^−^ described for simple phenolic acids, observed here at *m*/*z* 109 and *m*/*z* 123, respectively (Table 2). The UV maxima and the MS^2^ fragmentation patterns obtained were consistent with the chemical data previously described for the glycosylated phenolic acids or their aglycons [36,37,38,39,40]; thus, these compounds were proposed as protocatechuic acid hexose **[R12],** hydroxybenzoic acid hexose **[R18]**, and vanillic acid hexose **[R26]**.

**Flavonoids.** Five discriminant compounds of root or leaf extracts were assigned to the flavonoids class. The peak **[L29]** presented a UV spectrum expected for a flavonol (λ_max_ at 256, 265 sh, 295 sh, 353 nm) and showed in negative MS^2^ analysis a precursor ion [M-H]^−^ at *m*/*z* 477.0669, giving a fragment ion at *m*/*z* 301 (loss of 176 uma), a characteristic ion of the quercetin genin after cleavage of a glucuronic acid moiety. The two other major fragment ions at *m*/*z* 179 and 151 were consistent with fragmentation described for the quercetin moiety; thus, **[L29]** was identified as quercetin-3-*O*-glucuronide [33,34,40]. For the peak **[L30]**, λ_max_ values (228, 290, 335 sh) indicated a flavanonol or flavanone compound. In MS analysis, it was observed as a precursor ion at *m*/*z* 449.1085, yielding major fragment ions at *m*/*z* 303, 285, and 151 corresponding to [(M-C_6_H_10_O_4_)-H]^−^ (loss of a rhamnose moiety, 146 uma), [(M-C_6_H_10_O_4_-H_2_O)-H]^−^, and to a Retro–Diels Alder (RDA) cleavage of the flavonoid genin, respectively. All these results (Table 2), in accordance with the literature [33,41,42,43], led to identifying **[L30]** as astilbin (taxifolin-3-*O*-rhamnoside). Some flavan-3-ols were detected in the roots extracts: the UV and MS data of the peak **[R30]** (Table 1) indicated a catechin isomer [44,45]. Comparison to authentic standards analyses revealed that **[R30]** and **[R42]** were catechin and epicatechin, respectively; the latter is not a discriminant compound. The peak **[R55]** was another flavan-3-ol derivative: it was observed as [M-H]^−^ at *m*/*z* 441.0825, dissociated in MS^2^ experiment in major product ions at *m*/*z* 289, 245, 169, and 125, previously described to correspond to fragments as (epi)catechin, decarboxylated (epi)catechin, gallic acid, and decarboxylated gallic acid, respectively [45,46]. On the basis of this spectral data consistent with the literature, **[R55]** was tentatively identified as (epi)catechin monogallate. In the peak **[R48]**, a flavan-3-ol polymer was detected: it was observed as a precursor ion [M-H]^−^ at *m*/*z* 729.1455, generating in MS^2^ experiment major product ions at *m*/*z* 577 ([M-H-152]^−^, loss of a galloyl group), *m*/*z* 441 ([M-H-288]^−^, loss of an (epi)catechin unit), *m*/*z* 407 ([M-H-152-18]^−^, resulting from RDA fragmentation and water elimination or loss of a galloyl group and water), and *m*/*z* 289 ([M-H-441]^−^, loss of an (epi)catechin gallate moiety). These MS data were in accordance with the literature [45,47,48], and consequently, this compound in peak **[R48]** was assigned to a procyanidin dimer monogallate.

**Stilbenoids.** Two root discriminant compounds were assigned to the stilbenoids class. First, the UV spectrum of peak **[R47]** exhibited λ_max_ (224, 302, and 322 nm), similar to the ones of piceatannol [49]. MS analyses revealed a precursor ion at *m*/*z* 405.1193, showing in MS^2^ a neutral loss of 162 uma (loss of hexose), leading to the major product ion at *m*/*z* 243.0647, corresponding to piceatannol. The other fragment ions observed (Table 1) were concordant with those previously described in the fragmentation pattern of piceatannol [50]. Thus, **[R47]** was identified as a piceatannol hexoside (like astringin). Secondly, the peak **[R80]** showed a signal at *m*/*z* 677.1816, consistent with a compound with the molecular formula C_42_H_30_O_9_ (theoretical [M-H]^−^ ion at *m*/*z* 677.181706, ∆ppm −0.2, Table 1). The MS^2^ product ions obtained here for this compound (Table 1) were coherent with those exhibited in the MS^2^ spectrum previously published for α-viniferin [51]. The peak **[R80]** was thus proposed to be assigned to α-viniferin.

**Hydrolyzable tannins.** A wide range of compounds was assigned to belong to the class of hydrolyzable tannins in both root and leaf extracts. Among the discriminant compounds, the peaks **[R8]** and **[L27 = R58]** showed UV-vis spectra with λ_max_ (222 and 274 nm; 252, 292 sh, 302, 354 sh, and 368 nm, respectively), consistent with the ones previously described for gallic and ellagic acids, respectively [52,53]. Analyses of authentic standards confirmed these identifications based on retention times, UV-vis, and MS spectra. Other discriminant compounds were assigned to gallotannins, structures based on a glucose core esterified with gallic acid residues, which led in MS^2^ experiment to common losses of 152 or 170 uma (gallic acid moieties) and of 162 or 180 uma (hexose part). Compounds detected in peaks **[R7 = L3]**, **[L10]**, and **[R51 = L20]** as [M-H]^−^ ions at *m*/*z* 331.0673, *m*/*z* 483.0788, and *m*/*z* 635.0882, respectively, showed these types of product ions in MS^2^ spectra (Table 1 and Table 2), which were consistent with those previously described for galloyl-hexose, di-*O*-galloyl-hexose, and tri-*O*-galloyl-hexose, respectively [38,54,55]. At last, a series of twelve compounds were tentatively assigned to ellagitannins **[L16; L17 = R48; L19 = R49; L25 = R57; L28 = R38; R66; R58]**, some were present both in root and leaf extracts. These ellagitannins are constituted of a polyol core (usually glucose) esterified with hexahydroxydiphenic acid(s) (HHDP), ellagic acid, and sometimes with gallic acid residues and can be simple monoesters to complex polyesters. The fragmentation pattern of ellagitannins has been described as less clear than that of gallotannins because of their wide structural variability due to the diversity of possible linkages between the residues constituting them [46]. In negative ionization mode, an important diagnostic ion of ellagitannin is the one of ellagic acid, observed at *m*/*z* 300.99 [55], and the presence of an ion at *m*/*z* 169 reveals galloyl units in the structure. All the discriminant peaks annotated as being ellagitannins presented these characteristic diagnostic product ions, but the other MS^2^ data did not allow us to go further in the structural determination. As described before, some of the detected ellagitannins, in peaks **[L16; L17 = R48],** were observed as [M-2H]^2−^ ion in addition to the deprotonated molecule [M-H]^−^ [40]. In the peaks **[L25]** and **[R57]**, the same series of three ions (*m*/*z* 779, *m*/*z* 797, and *m*/*z* 815) were observed with a difference of 18 uma between them and similar fragmentations, corresponding to a series of ellagitannins more or less hydroxylated.

#### 2.3.2. Discriminant Compounds in Roots

**Figure 7:**
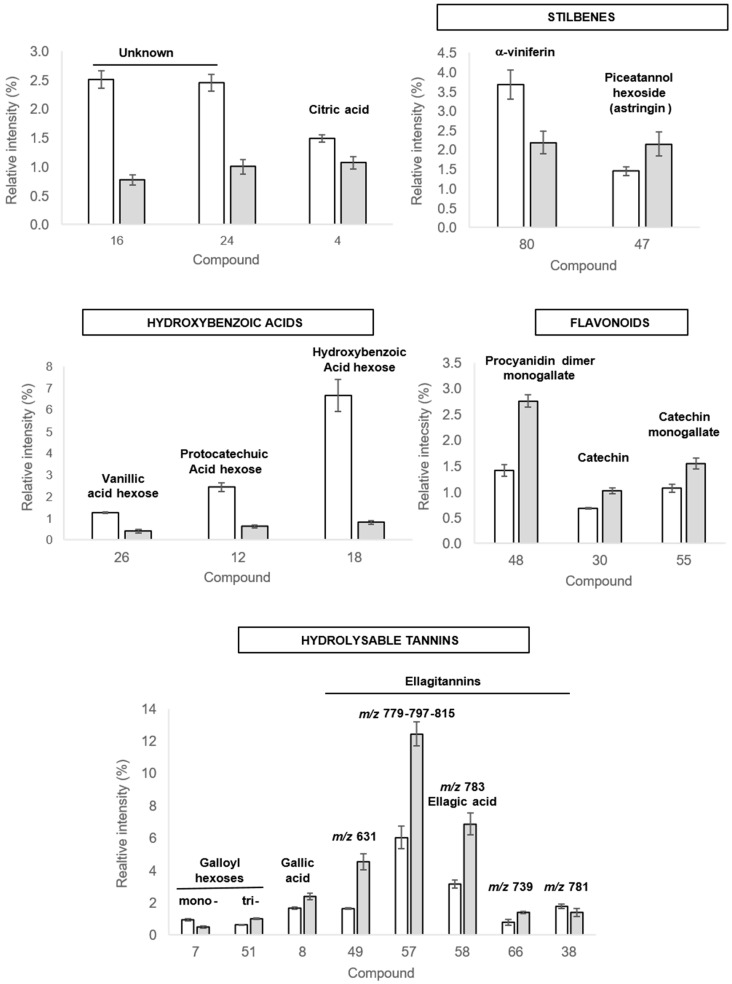
Grapevine root secondary metabolites relative abundances in control (white) and *Paraburkholderia phytofirmans* PsJN-bacterized (grey) plants. Only the discriminant compounds (VIP > 1) with significant differences between the two treatments (Student’s t-test, *p* < 0.05) are represented. The compound numbers indicated are those listed in Table 1. The relative abundance values shown are means ± standard deviation (control, *n* = 5; PsJN, *n* = 8) for a representative experiment of two independent experiments.

The identified discriminant compounds highlighted to be modified by PsJN in roots belong to four major types of phenolic compounds (Table 1). The level of several glycosylated **hydroxybenzoic acids** (vanillic acid hexose **[R26]**, protocatechuic acid hexose, i.e., 3, 4 di-hydroxybenzoic acid hexose **[R12],** and hydroxybenzoic acid hexose **[R18]**) showed a significant intensity decrease in grapevine roots in response to PsJN (3, 4, and 8-fold, respectively) (Figure 7). Among **flavonoids** compounds, one proanthocyanidin (procyanidin dimer monogallate **[R48]**) and two flavan-3-ols (catechin **[R30]** and catechin monogallate **[R55]**) were accumulated in response to PsJN (Figure 7). Among **hydrolyzable tannins**, monogalloyl hexose **[R7]** and an ellagitannin (*m*/*z* 781) **[R38]** were less observed in bacterized plantlets compared to control ones (Figure 7). On the contrary, trigalloyl hexose **[R51]**, gallic acid **[R8]**, a compound similar to castallin/vescalin **[R49]**, ellagic acid **[R58]**, and ellagitannins *m*/*z* 779-797-815 **[R57]** and *m*/*z* 739 **[R68]** were accumulated in response to PsJN. Finally, we identified two **stilbenoids***,* showing a contrasting modification in the presence of the bacterium: α-viniferin (a cyclic dehydrotrimer of resveratrol) **[R80]** was less present, whereas piceatannol hexoside (astringin) **[R47]** was accumulated in response to PsJN.

#### 2.3.3. Discriminant Compounds in Leaves

**Figure 8:**
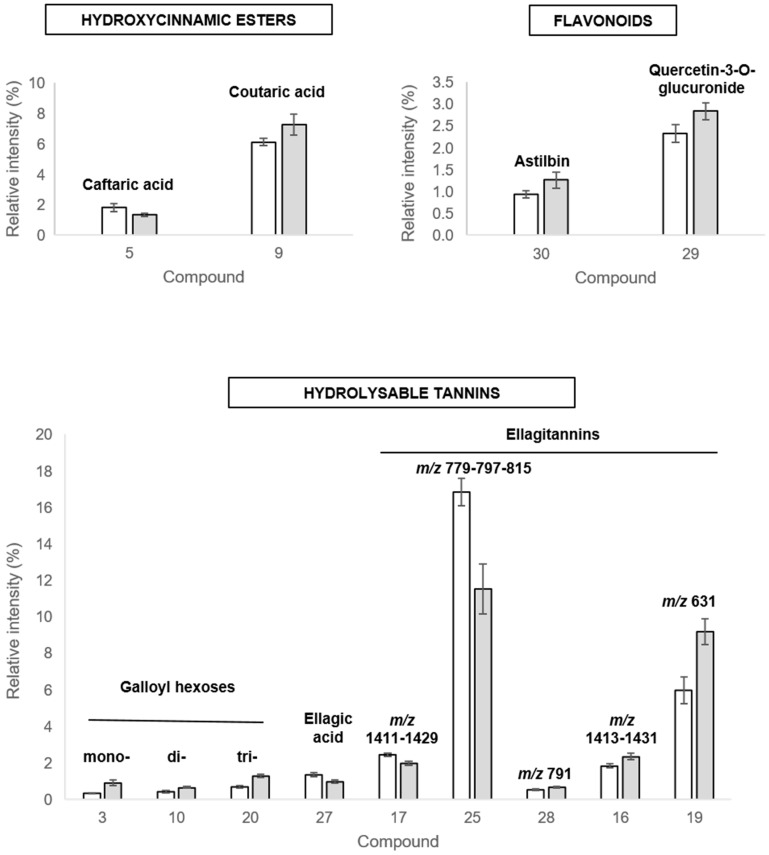
Grapevine leaves secondary metabolites relative abundances in control (white) *Paraburkholderia phytofirmans* PsJN (grey) plants. Compound numbers are indicated within brackets below retention times. Only the discriminant compounds (VIP > 1) with significant differences between the two treatments (Student’s t-test, *p* < 0.05) are represented on this histogram. The compound numbers corresponded to compounds listed in Table 2. The relative abundance values shown are means ± standard deviation (*n* = 9) for a representative experiment of two independent experiments.

For the leaves, 13 discriminant metabolites were identified (Table 2, Figure 8) that belong to three major types of phenolic compounds. Two **hydroxycinnamic esters** presented a contrasted modification in response to the PGPR: the level of caffeoyl tartaric acid (caftaric acid) **[L5]** tended to decrease, whereas the level of *p*-coumaroyl tartaric acid (coutaric acid) **[L9]** was increased in bacterized leaves compared to control ones. A significant accumulation of two **flavonoids**, one flavanonol (astilbin) **[L30]**, and one flavonol (quercetin-3-*O*-glucuronide) **[L29]** was observed in leaves after PsJN bacterization. Nine metabolites related to **hydrolyzable tannins** pathway were highlighted in our approach. Mono-, di-, and trigalloyl hexoses **[L3-L10-L20]** tended to increase in bacterized plantlets (Figure 8). Ellagic acid **[L27]** and two ellagitannins (*m*/*z* 1411-1429 **[L17]** and *m*/*z* 779-797-815 **[L25]**) diminished in response to PsJN, whereas three other ellagitannins (*m*/*z* 791 **[L28]**, *m*/*z* 1413-1431 **[L16]**, *m*/*z* 631, similar to castallin/vescalin **[L19]**) increased. We could notice that the same compounds were annotated in roots **[R57]** and leaves **[L25]**. The PsJN inoculation strongly affected the level of these compounds in an opposite manner between the two organs: accumulation in roots and depletion in leaves.

### 2.4. Effect of Some Accumulated Phenolic Compounds on Botrytis cinerea

**Figure 9:**
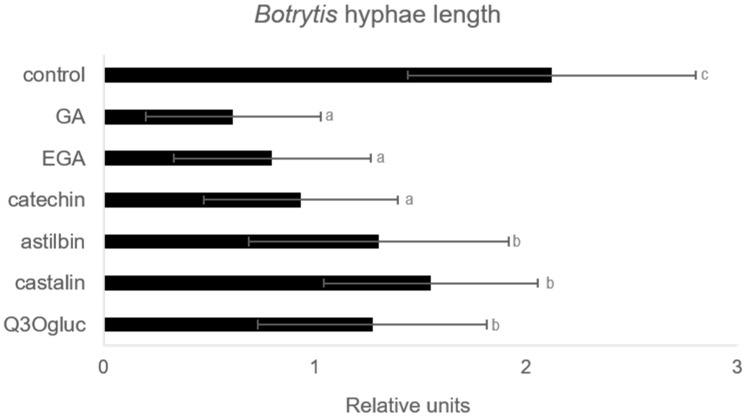
Effect of molecules on *Botrytis cinerea* development. Conidia were placed in growth medium supplemented with solutions of molecules at 0.1 mg/mL. The length of germ tubes was observed by inverted light microscopy 8 h later and measured using Image J. GA, gallic acid; EGA, ellagic acid; Q3Ogluc, quercetin-3-*O*-glucuronide. Different letters above each bar indicate significant differences (*p <* 0.05), as determined by Tukey’s analysis.

We showed in this study that gallic acid, ellagic acid, catechin, astilbin, a compound similar to castalin, and quercetin-3-*O*-glucuronide were accumulated in grapevine roots or leaves in response to PsJN. Since these compounds were previously described for their antimicrobial effect, we tested their ability to inhibit the development of a well-known grapevine pathogen, *Botrytis cinerea*. As shown in Figure 9 and Supplemental Figure S3, these six molecules applied at 0.1 mg/mL had a direct antimicrobial effect in vitro on the fungal spore germination.

## 3. Discussion

During plant-PGPR interactions, modifications in phenolic metabolite profiles have been previously observed [7,56]. Moreover, the induction of secondary metabolites synthesis was reported to be correlated with plant growth promotion and induction of disease resistance [57]. A PGPR strain, *P. phytofirmans* PsJN, was previously described as a grapevine growth promoter and inducer of plant resistance against *Botrytis cinerea* [25]. Accumulation of total phenolic contents following PsJN inoculation in plantlets was previously reported [15], and we observed a red color of grapevine plantlets after root-inoculation with this beneficial bacterium. We investigated in the present study the impact of *P. phytofirmans* PsJN on grapevine phenolic secondary metabolites by monitoring gene expression and profiling of plant secondary metabolites from both roots and leaves of control and PsJN-inoculated grapevine plantlets.

At the root level, our metabolomic approach showed the involvement of the glycosylation process in the response of grapevine to PsJN inoculation (decrease of glycosylated hydroxybenzoic acids derivatives). The glycosylation usually changes the bioactivity, solubility, subcellular localization, and binding property of phenolic compounds [58]. The less content of glycosylated forms could be due to the secretion of aglycon forms as plant defense mechanisms against microorganisms. Indeed, the aglycon form of these compounds was previously described for their efficient antimicrobial activity against Gram-negative and -positive bacteria [59], as well as for their action against biofilm [60]. Moreover, hydroxybenzoic acids may be involved in signaling, particularly in the induction of defense and stress responses [9]. Alternatively, the decrease of glycosylated phenolics could be the consequence of a metabolic split, if the compounds from the shikimate pathway are mainly used for the synthesis and accumulation of hydrolyzable tannins. These important bioactive polyphenols identified in our study are divided into tannins of gallic and ellagic origin. These are well-known to have an antioxidant effect [61], possibly correlated with the oxidative stress response. Indeed, most of the ellagic acid content of plant cells exist in the vacuoles as water-soluble ellagitannins, and it is thought to play a role in plant defense against pathogen attacks [61]. From the ecological point of view, the fact that inoculation by PsJN leads to an accumulation of these compounds may help the bacterium to efficiently establish in the grapevine rhizosphere through the use of plant metabolic abilities to manage its niche. Hence, it will be interesting to test the effect of this PGPR on the root microbiome, including pathogens.

**Figure 10:**
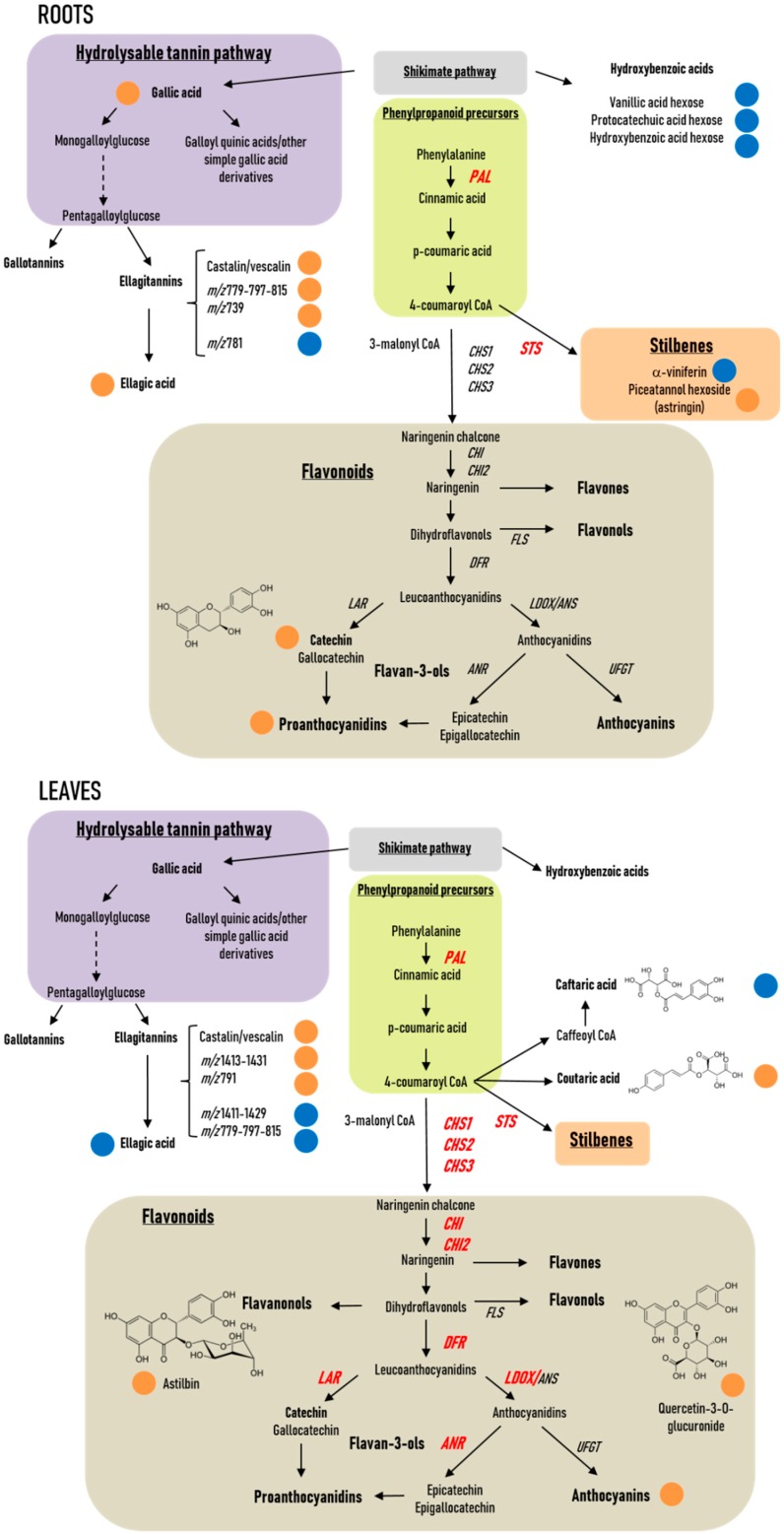
Overview of PsJN effect on grapevine phenolic metabolism. Genes up-regulated by PsJN are indicated in red. Metabolites, blue: control > PsJN; orange: control < PsJN.

In roots, concomitant activation of both *PAL* and *STS* genes was observed in response to PsJN from the earliest stages of colonization (1dai) (Figure 4 and Figure 10). Such coordinated induction of *PAL* and *STS* was previously mainly observed in response to pathogens, *Botrytis cinerea* [62] or *Eutypa lata* [63], and elicitor treatment, BTH [64] or MeJA [65]. *PAL* and *STS* were reported to be major genes in the resistance of *Vitis vinifera* [66]. Stilbenoids could act as phytoalexins and could be produced de novo in plants to protect against pathogens. They are also able to exert other biological activities [67]. Here, we identified two stilbenoids, showing a contrasted modulation in the presence of PsJN. Actually, α-viniferin, a cyclic dehydrotrimer of resveratrol, was down-modulated, whereas piceatannol hexoside (astringin) was up-modulated (Figure 10). Piceatannol was described in grape [49] and showed potent biological activities in vitro, including antioxidant and pro-apoptotic activities [68,69], often demonstrated as more efficient than resveratrol [70]. Our results indicated that PsJN might favor the glycosylated storage form of piceatannol rather than the active form (α viniferin), preparing the plant against potential biotic or abiotic stresses. In this study, we also observed that some flavonoids (proanthocyanidin and flavan-3-ols) were accumulated in response to PsJN (Figure 10). These defense compounds are major contributors to the biological activities in products derived from grapes [71,72]. They are synthesized in the cytoplasm, induced upon pathogen attack, or exuded into the soil [73] and are known to inhibit a range of root pathogens [74,75]. Moreover, exuded flavonoids are well-known signals, influencing the ability of bacteria to colonize the rhizosphere (i.e., molecular signalization in *Rhizobium* legume interaction). In addition to flavonoids, phenolic acids and ellagitannins are accumulated in roots following PsjN inoculation. They are also known for their high antioxidant and free scavenging activities [76,77,78]. Moreover, the presence of galloyl groups in such compounds has an impact on biological properties as iron chelation or antioxidant activity [79].

In leaves, as in roots, *PAL* and *STS* were concomitantly induced in response to PsJN. However, *PAL* and *STS* expression in leaves appeared 7 days when bacteria were detected in the aerial parts (Supplemental Figure S1). This indicated that *PAL* and *STS* response to PsJN inoculation did not seem to be systemic. Conversely, in leaves, we observed an overexpression of all genes related to the flavonoid biosynthesis pathway, and that from the 4th day following PsJN inoculation when the bacterium was not detected yet in leaves. These results at the gene expression level were in accordance with results obtained at the metabolomics level. Indeed, we measured the accumulation of the flavonol quercetin-3-*O*-glucuronide, and also the cinnamate ester coutaric acid, in response to PsJN. This could be correlated to the increase of anthocyanins and reddish coloration of leaves for inoculated plants. Actually, flavonols (quercetin-3-*O*-glucuronide) act as copigments for anthocyanins [80] and coutaric acid as a vehicle for anthocyanins [81]. Anthocyanins are soluble secondary metabolites that accumulate in the vacuoles of epidermal cells of the leaves and grape berries [82,83]. Anthocyanins confer major ecological and physiological benefits for plants, and their accumulation can be induced by various biotic or abiotic agents. Mono-glycosylated anthocyanins are able to promote increased levels of cellular glutathione and activate free radical detoxification enzymes, thereby protecting rat liver cells against H_2_O_2_-induced oxidation [84,85]. In addition to their classic roles in color modification and health benefits, the biocontrol research has demonstrated that genetically modified tomato fruits overproducing anthocyanins have reduced susceptibility to *Botrytis cinerea* [86]. Also, the radical scavenging capacity of anthocyanins can effectively slow the increase in reactive oxygen species (ROS) during tomato infection by *B. cinerea*, and thus reduce fruit infection [87]. In this context, by favoring the increase of the anthocyanin content in grapevine plantlets, the bacterium PsJN could give it better resistance to future attacks by pathogenic microorganisms. Actually, we showed a tendency of individual compounds, accumulated in root or leaves following PsJN inoculation, to interfere with *B. cinerea* development. Indeed, catechin and quercetin-3-*O*-glucuronide were described as potent stilbene oxidase inhibitors, reducing fungal defense, and so its growth [88].

In conclusion, we showed that PsJN was able to induce locally (in roots) and systemically (in leaves) the expression of key enzymes of phenolic metabolism. Metabolomic analyses showed that hydrolyzable tannins and flavonoids were accumulated in grapevine plantlets following PsJN inoculation. These compounds are well-known for their antimicrobial effect, so we tested their impact on *Botrytis cinerea* development and showed an inhibitory effect on fungal spore germination. We demonstrated previously that PsJN is able to protect against *B. cinerea* [25] by a direct antifungal effect, combined with priming of defense mechanisms. Our findings on the impact of PsJN on phenolic metabolism allowed to suggest a supplementary biocontrol mechanism developed by this PGPR to fight against pathogens.

## 4. Material and Methods

### 4.1. Chemicals

All chemicals were of HPLC-MS grade and used as received. Acetonitrile, water, and formic acid for UHPLC-MS QTOF analyses were purchased from Fisher Scientific (Optima^®^ grade, Fisher Scientific, Geel, Belgium). The standard compounds used, castalin, astilbin, and quercetin-3-*O*-glucuronide, were obtained from Merck (Darmstadt, Germany); citric acid from LIPHA (Lyon, France); gallic acid from Fluka (Buchs, Switzerland); ellagic acid, catechin, and epicatechin from Sigma–Aldrich (St Louis, MO, USA).

### 4.2. Plant Material

Plantlets of *Vitis vinifera* cv. Chardonnay (clone 7535) were micro-propagated by nodal explants, grown on 15 mL of agar medium in 25 mm-culture tubes, as described in [15]. Cultures were performed in a growth chamber under white fluorescent light (200 μmol/m^−2^ s^−1^), with 16 h/8 h day/night photoperiod at a constant temperature of 26 °C.

### 4.3. Microorganisms

*Paraburkholderia phytofirmans* strain PsJN tagged with GFP was cultivated in King’s B liquid medium [89] supplemented with kanamycin (50 μg/mL) for 24 h with agitation (180 rpm) at 28 °C. Bacteria were collected after centrifugation at 4500 g at 4 °C for 15 min and suspended in phosphate buffer saline (PBS 10 mM, pH 6.5). The concentration of bacteria was determined by spectrophotometry (600 nm) and adjusted to 10^9^ CFU/mL with PBS (OD = 0.8).

### 4.4. Inoculation of Vitroplantlets with P. phytofirmans Strain PsJN

Roots of 4-weeks-old grapevine plantlets were inoculated with 200 μL of bacterial inoculum (10^9^ CFU/mL). For gene expression analyses, leaves and roots from control and bacterized plants were sampled 1, 4, and 7 days after bacterization and immediately frozen in liquid nitrogen and stored at −80 °C. For phenolic compounds analyses, control and bacterized plantlets were transferred one week after bacterization aseptically into sterile Magenta boxes containing 60 g of soil for 3 days. Leaves and roots were sampled, pooled (9 plantlets/pool), immediately frozen in liquid nitrogen, freeze-dried 24 h, ground, and stored at −80 °C.

### 4.5. Rhizoplane and Endophytic Colonization

Rhizoplane and endophytic colonization were followed, as described in [25]. Briefly, for colonization of *P. phytofirmans* PsJN in the roots, the samples were removed from the soil and vortexed (240 rpm) with PBS (10 mM, pH 6.5) for approximately 1 min. The homogenate was serially diluted in 10 fold steps and cultured on King’s B medium plates (in triplicates), supplemented with kanamycin (50 mg/mL). For endophytic colonization, roots were surface sterilized with 70% ethanol for 1 min, followed by 1% commercial bleach and a 0.01% Tween 20 solution for 1 min, and then washed four times in distilled water (1 min each time). Leaves were surface sterilized with 1% commercial bleach and a 0.01% Tween 20 solution for 3 min, and then washed four times in distilled water (1 min each time). The samples were then ground with 1 mL of PBS. The homogenate was serially diluted in 10 fold steps and cultured on King’s B medium plates (triplicates), supplemented with kanamycin (50 mg/mL). The bacterial colonies were counted after 3 days of incubation at 28 °C.

### 4.6. RNA Extraction and Real-Time Quantitative RT-PCR

For each sample, 50 mg of leaves and roots were ground in liquid nitrogen. Total RNA was isolated using Plant RNA (Eurobio, Les Ulis, France)), and 150 ng was used by reserve transcription using the Verso cDNA Synthesis Kit (Thermo Scientific,) according to the manufacturer’s instructions. The transcript levels were determined by real-time quantitative PCR using the CFX 96 T^M^ Real-Time System (Biorad, Hercules, CA, USA) and the SYBR Green Master Mix PCR kit as recommended by the manufacturer (Applied Biosystems). PCR conditions were 95 °C for 15 s (denaturation) and 60 °C for 1 min (annealing/extension) for 30 cycles. Reactions were carried out, as described in [25]. The specific primers used in this study are listed in Supplemental Table S1.

### 4.7. Extraction of Phenolic Compounds

For anthocyanin extraction, 50 mg leaf powder was mixed in an Eppendorf tube (1.5 mL) with 625 µL of 1% HCl in methanol (*v*/*v*). The samples were incubated overnight, under stirring, in the dark at 4 °C. Then, 375 µL of water and 375 µL of chloroform were added and mixed with the extract. Chloroform was added to remove the chlorophyll by mixing, and the solution was centrifuged (15,000× *g* for 2 min). The anthocyanin contained in the aqueous phase was recovered, and the absorbance at 535 nm was determined by spectrophotometry. The means ± standard deviations originated from two independent experiments; each replicate consisted of a pool of 70 plantlets.

For the metabolomic study, extractions of phenolics were performed on 15 mg of a freeze-dried leaf or root biomass. Dried powders were firstly extracted twice with 1 mL of MeOH 80% and, secondly, extracted once with 1 mL of pure MeOH, with vortex homogenization, 15 min of sonication (Bransonic^®®^ ultrasonic cleaner 2510EDTH) at room temperature and centrifugation (20,238× *g*, 10 min) to keep the extract solution for each step of extraction. The combined extracts were centrifuged again (20,238× *g*, 10 min), and the obtained supernatant, particle-free, was evaporated to dryness at 30 °C in a concentrator (CentriVap Concentrator Labconco, USA) to constitute the dried crude extract. These dried extracts obtained were weighed and dissolved in MeOH 60% at 2.5 mg·mL^−1^ for the leaves, and 10 mg·mL^−1^ for the roots.

For these phenolic compounds analyses, two independent biological repeats were performed. Each repeat included 70 seedlings per treatment. We realized 9 technical repetitions for the leaves and 4 technical repetitions for the roots. For each biological repeat and each plant part, a quality control (QC) sample containing all the replicate samples was prepared by mixing an equivalent volume of each replicate sample. These QC samples of each plant part were initially analyzed and were then injected after every 10 samples in the run sequence to monitor the repeatability of the analysis. The samples were stored until analysis at −20 °C.

### 4.8. Analyses by UHPLC-UV/DAD-MS ESI QTOF

Phenolic compounds analyses were performed on a UHPLC Agilent 1290 coupled to a UV-vis Diode Array Detector (Agilent 1290 Infinity series) and an Accurate-Mass Q-TOF 6530 spectrometer (Agilent Technologies, County of Santa Clara, CA, USA) with an electrospray ionization source (ESI, Agilent Jet Stream Technology, County of Santa Clara, CA, USA). The equipment was managed by using the Mass Hunter Workstation Data Acquisition software B.07.00 (Agilent Technologies, County of Santa Clara, CA, USA). For chromatographic separation, a Poroshell 120 EC-C_18_ column (3 × 150 mm, particle size 2.7 μm, Agilent technologies, County of Santa Clara, CA, USA), equipped with a Poroshell C_18_ pre-column (3 × 5 mm, particle size 2.7 μm, Agilent technologies, County of Santa Clara, CA, USA), was used at a constant temperature of 60 °C and with injections of 3 μL of sample solution. The mobile phase used was a mixture of acetonitrile (Optima^®®^ grade, Fisher Scientific, Geel, Belgium) and acidified water (0.4% formic acid) at a flow rate of 1 mL/min. For the leaves, the gradient was as follow: starting at 3% of CH_3_CN for 1 min, increasing to 17% in 7 min, then enhancing to 60% in 1 min before increasing to 80% in 3 min, then going to 100% in 1 min and maintained for 2 min, before returning to the starting conditions in 0.5 min and equilibrating for 1.5 min. For the roots extracts analyses, the gradient was as follow: starting at 1% of CH_3_CN for 1 min, increasing to 14% in 10 min and maintained for 1 min, enhancing to 45% in 8 min, then going to 100% in 1 min and maintained for 2 min, before returning to the starting conditions in 0.5 min and equilibrating for 2.5 min.

The QToF-MS was used with its ESI source in negative ionization mode under the following parameters: carrier and auxiliary gas temperature at 320 °C and 350 °C, respectively, and with flow rates at 11 L/min, with voltages of capillary, nozzle, and fragmentor at 3500 V, 500 V, and 150 V, respectively. The acquisition of the spectra was carried out in a range of *m*/*z* 50–2000. For the characterization of compounds, complementary MS/MS analyses were performed in negative and positive ionization mode with different collision energies applied (10, 20, 30, or 40 V) in autoMS/MS mode. Data reprocessing was done using Mass Hunter Qualitative Analysis Software B.07.00 (Agilent Technologies). The comparison of phenolic compounds profiles was performed on chromatographic peak data integrated at 280 nm (a wavelength allowing the detection of a wide range of secondary metabolites and especially phenolic compounds). A data matrix was created with the absolute area under curve (AUC) values of all peaks in all samples, applying the necessary alignments. Normalization of the matrix data was performed by calculating relative areas in percentage per sample. Statistical analyses were performed on this data matrix. Identification/annotation of discriminating metabolites was attempted based on the UV-vis spectrum and the positive and negative MS and MS/MS spectra. These data were compared to literature data, and the following public databases were also consulted: SciFinder Scholar (https://scifinder.cas.org), ChemSpider (http://www.chemspider.com), and KNApSAcK Core System (http://www.knapsackfamily.com/knapsack_jsp/top.html). When possible, comparisons with authentic standards allowed confirming metabolite identity.

### 4.9. Spore Germination Assay

*B. cinerea* spore germination with the different molecules (0.1 mg·mL^−1^) was assessed in 96-well microplates. *B. cinerea* was collected in potato dextrose broth (PDB) and was added in each well to a final concentration of 5000 spores, in triplicate, in a total volume of 100 μL. The plates were incubated at 20 °C in the dark. Germ tube growth was observed 8 h after challenge using an inverted light microscope (Leica, Wetzlar, Germany).

### 4.10. Statistical Analysis

All statistical analysis and plots were performed with the open source software R version 3.2.2 (RCoreTeam, 2015, R Foundation for Statistical Computing, Vienna, Austria) using “agricolae”, “car” “ggplot2” and “mixomics” packages (downloaded in 2016). To compare phenolic compounds profiles, a principal component analysis (PCA) was performed on uni variance scaled data; scores and loadings of the first two components were plotted. A PLS-DA (partial least squares discriminant analysis) was also performed on this dataset to obtain the list of compounds whose VIP value was ≥1 (variable importance for the projection). The VIP allows us to prioritize the variables according to their explanatory importance in the observed discrimination. This allows us to quickly identify which are the most important explanatory variables. Shapiro–Wilk test (α > 0.05) was used for the normality test, and the F test (α > 0.05) used for the homogeneity of variances test. The student’s t-test was used when values showed normal distribution. For data that did not have a normal distribution, Welch’s student test with correction was performed. The Wilcoxon–Mann–Whitney test was applied for non-parametric data.

## Figures and Tables

**Table 1:** Retention time and spectral data of the discriminant compounds annotated in root extracts by UHPLC-UV/DAD-ESI-MS-QTOF.

Peaks	Rt (min)	λmax (nm)	UHPLC-MS QTOF Analysis					UHPLC-MS/MS QTOF Analysis			Compound Annotation	References
			Ionization Mode	Observed Ions (*m*/*z*)	Ionic Species	Ion Formula	∆ppm	Collision Energy (V)	Precursor Ion (*m*/*z*)	Main Product Ions *m*/*z* (% Base Peak)		
**R4**	0.96	/	-	191.0191	[M-H]^−^	C_6_H_7_O_7_	−3.3	10	191	173(2) 129(5) **111(100)**	Citric acid *	[31,32]
			-	405.0285	[2M+Na-2H]^−^	C_12_H_14_O_14_Na	−0.4	20	405	191(97) 173(9) 129(4) **111(100)**		
**R7**	1.30	222 276	-	331.0665	[M-H]^−^	C_13_H_15_O_10_	−1.7	20	331	331(23) 271(16) 211(32) **169(100)** 151(36) 125 (22) 123(35)	Galloyl-hexose	[38,54]
**R8**	1.44	222 274	-	169.0136	[M-H]^−^	C_7_H_5_O_5_	−3.8	20	169	**125(100)**	Gallic acid *	[55]
**R12**	2.24	264 294	-	315.0718	[M-H]^−^	C_13_H_15_O_9_	−1.1	20	315	315(5) 255(7) 195(27) **153(100)** 109(32)	Protocatechuic acid hexose	[36,38]
			+	339.0695	[M+Na]^+^	C_13_H_16_O_9_Na	2.5					
**R16**	2.88	226 278	-	335.0975	[M-H]^−^	C_13_H_19_O_10_	−2.6	10	335	335(44) **289(100)** 161(38)	Unknown	
**R18**	3.26	262	-	299.0769	[M-H]^−^	C_13_H_15_O_8_	−1.1	20	299	299(5) 239(9) 179(47) 151(14) **137(100)** 119(14) 113(18) 101(21)	Hydroxybenzoic acid hexose	[36,40]
			+	323.0743	[M+Na]^+^	C_13_H_16_O_8_Na	1.7	20	323	323(27) **185(100)** 161(19)		
			+	623.1591	[2M+Na]^+^	C_26_H_32_O_16_Na	1.4	30	623	**323(100)** 185(43) 161(6)		
**R24**	3.83	225sh 254 308sh	-	497.0589	[M-H]^−^	C_20_H_17_O_15_	3.2	10	497	497(40) 479(74) **453(100)** 393(33) 298(25) 291(49) 273(32) **247(95)**	Unknown	
**R26**	4.46	224 266 294	-	329.0867	[M-H]^−^	C_14_H_17_O_9_	−3.4	30	329	209(25) 167(57) 152(39) **123(100)** 122(49) 108(51) 101(38)	Vanillic acid hexose	[36,37,39]
			+	353.0836	[M+Na]^+^	C_14_H_18_O_9_Na	−2.0	20	353	353(32) 193(31) 191(14) **185(100)**		
			+	683.1807	[2M+Na]^+^	C_28_H_36_O_18_Na	1.9	40	683	353(86) 191(15) **185(100)**		
**R30**	5.11	226 276	-	289.0716	[M-H]^−^	C_15_H_13_O_6_	−0.6	10	289	**289(100)** 245(34) 205(11) 203(10) 179(9) 125(10) 109(9)	Catechin *	[44,45]
**R38**	6.87	224 268	-	781.0533	[M-H]^−^	C_34_H_22_O_22_	0.4	30	781	763(38) 745(64) 735(15) 461(14) **300.99(100)** 299(88) 273(29) 229(23)	Ellagitannin *m*/*z* 781	
**R47**	8.49	224 302 322	-	405.1193	[M-H]^−^	C_20_H_21_O_9_	0.5	40	405	**243(100)** 241(23) 225(10) 201(52) 199(11) 175(15) 173(14) 159(54)	Piceatannol hexoside (astringin)	[49,50]
**R48**	8.62	226 273	-	729.1455	[M-H]^−^	C_37_H_29_O_16_	−0.8	20	729	729(57) 603(19) 577(29) 451(19) 441(28) **407(100)** 289(50) 169(15) 125(23)	Procyanidin dimer monogallate	[45,47]
			+	731.1614	[M+H]^+^	C_37_H_31_O_16_	1.0	20	731	731(10) 563(16) 443(28) 427(39) 409(79) 301(40) 290(19) 289(64) 287(28) 275(32) 273(40) 271(43) 259(19) 247(56) 163(36) 151(18) 139(23) 127(98) **123(100)**		
			-	705.0508	[M_1_-2H]^2−^	C_54_H_42_O_45_	0.5	20	705	705(91) **673(100) 300.99(67)**	Ellagitannin *m*/*z* 1411	
			-	1411.1077	[M_1_-H]^−^	C_54_H_43_O_45_	−0.3	40	705	**300.99(100)**		
			-	714.0512	[M_2−_2H]^2−^	C_54_H_44_O_46_	−6.4	10	714	**705(100)** 696(25) 691(47) 613(37) 601(17) 631(14) **300.99(11)**	Ellagitannin *m*/*z* 1429	
			-	1429.1149	[M_2_-H]^−^	C_54_H_45_O_46_	−2.7	30	714	613(50) **300.99(60)** 299(54) 273(79) **261(100)** 229(44) 215(55) 167(76) 123(35)		
**R49**	8.99	224 254sh 275 350sh	-	631.0577	[M-H]^−^	C_27_H_19_O_18_	0.02	30	631	613(70) 603(18) 577(23) 461(49) **445(100)** 443(49) 433(26) **300.99(89)** 299(79) 275(27) 273(30) 245(20) 231(31) 229(54) 169(33) 167(21) 123(27)	Ellagitannin *m*/*z* 631 (similar to castalin/vescalin)	[37]
**R51**	9.37	220 278	-	635.0881	[M-H]^−^	C_27_H_23_O_18_	−1.4	30	635	635(43) 483(24) 465(71) 423(8) 313(60) 295(10) 211(7) **169(100)** 125(13)	Tri-*O*-galloyl-hexose	[38,55]
**R55**	10.22	224 276	-	441.0825	[M-H]^−^	C_22_H_17_O_10_	−0.5	20	441	**289(44)** 271(6) 245(10) **169(100)** 125(25)	(*epi*)Catechin monogallate	[45,46]
**R57**	10.56	226 256 370sh	-	779.0372	[M-H]^−^	C_34_H_19_O_22_	−0.2	20	779	**751(100)** 733(37) 449(21) **300.99(78)** 299(56) 287(12) 275(12) 273(13)	Ellagitannin *m*/*z* 779	
			-	797.0484	[M-H]^−^	C_34_H_21_O_23_	0.6	20	797	779(18) **751(100)** 733(36) 449(18) 316(17) **300.99(92)** 299(74) 291(24) 273(57) 271(19) 247(19)	Ellagitannin *m*/*z* 797	
			-	815.0589	[M-H]^−^	C_34_H_23_O_24_	0.5	20	815	797(21) 779(27) **751(100)** 733(43) 725(20) 709(13) 449(13) 435(16) 317(13) **300.99(87)** 299(51) 291(45) 289(15) 273(60) 247(45)	Ellagitannin *m*/*z* 815	
**R58**	10.67	224 278 365sh	-	783.0689	[M-H]^−^	C_34_H_23_O_22_	0.3	40	783	765(75) 613(18) 597(33) 445(18) 427(22) **300.99(97)** 299(57) 275(98) 273(86) 271(19) 247(26) **229(100)** 169(57) 167(39) 123(40)	Ellagitannin *m*/*z* 783	
			-	300.9982	[M-H]^−^	C_14_H_5_O_8_	−2.6	30	301	**300.99(100)** 300(25) 283(26) 257(10) 245(17) 229(32) 201(24) 185(19) 173(21) 145(22) 129(8)	Ellagic acid *	[40,55]
**R66**	13.82	220 274 357sh	-	739.0786	[M-H]^−^	C_34_H_23_O_20_	−0.3	30	739	**721(100)** 569(13) 443(12) **300.99(27)** 299(13) 273(43) 229(39) 169(11) 166(38) 123(12)	Ellagitannin *m*/*z* 739	
**R80**	17.38	196 224 284	-	677.1816	[M-H]^−^	C_42_H_29_O_9_	−0.2	30	677	571(41) 529(33) 528(19) 501(72) 500(66) 477(21) 463(72) 449(18) **437(100)** 436(54) 435(48) 422(21) 407(18) 395(24) 394(20) 331(37) 330(38)	α-viniferin	[51]
			-	713.1589	[M+Cl]^−^	C_42_H_30_O_9_Cl	0.7					
			-	1355.3754	[2M-H]^−^	C_84_H_59_O_18_	3.5					
**R80**	17.38	196 224 284	+	679.196	[M+H]^+^	C_42_H_31_O_9_	−0.4	40	679	585(41) 491(25) 453(50) **359(100)** 345(83) 343(25) 331(42) 227(22) 215(24) 199(62) 121(45) 107(49)	α-viniferin	[51]
			+	701.1785	[M+Na]^+^	C_42_H_30_O_9_Na	0.4					

* annotation confirmed by comparison to authentic standard analysis.

**Table 2:** Retention time and spectral data of the discriminant compounds annotated in leaf extracts by UHPLC-UV/DAD-ESI-MS-QTOF.

Peaks	Rt (min)	λmax (nm)	UHPLC-MS QTOF Analysis					UHPLC-MS/MS QTOF Analysis			Compound Annotation	References
			Ionization Mode	Observed Ions (*m*/*z*)	Ionic Species	Ion Formula	∆ppm	Collision Energy (v)	Precursor Ion (*m*/*z*)	Main Product Ions *m*/*z* (% Base Peak)		
**L3**	0.96	222 278	-	331.0673	[M-H]^−^	C_13_H_15_O_10_	0.7	20	331	331(55) 271(33) 211(53) **169(100)** 151(35) 125(63) 123(36) 101(26)	Galloyl-hexose	[38,54]
**L5**	2.55	224 250sh 298sh 322	-	311.0411	[M-H]^−^	C_13_H_11_O_9_	0.8	20	311	179(21) **149(100)** 135(19) 103(7)	Caffeoyl-tartaric acid (caftaric acid)	[33,34]
			-	333.0229	[M+Na-2H]^−^	C_13_H_10_O_9_Na	0.3					
**L9**	3.64	232 295sh 312	-	295.0457	[M-H]^−^	C_13_H_11_O_8_	−0.8	20	295	**163(100)** 149(6) 119(92) 112(8)	Coumaroyl-tartaric acid (coutaric acid)	[33,34]
			-	317.0279	[M+Na-2H]^−^	C_13_H_10_O_8_Na	0.05					
			-	613.0794	[2M+Na-2H]^−^	C_26_H_22_O_16_Na	−2.9					
**L10**	3.92	224 278	-	483.0788	[M-H]^−^	C_20_H_19_O_14_	1.6	30	483	331(8) 313(17) **271(100)** 241(10) 211(32) 169(97) 125(18)	Di-*O*-galloyl-hexose	[38,55]
**L16**	5.59	222 274	-	706.0524	[M_1_-2H]^2−^	C_54_H_44_O_45_	−8.3	20	706	633(38) 631(61) 615(40) **613(100)** 611(40) 601(40) 419(41) **300.99(36)** 247(39) 245(36) **169(53)**	Ellagitannin *m*/*z* 1413	
			-	1413.1404	[M_1_-H]^−^	C_54_H_45_O_45_	11.7	40	706	**300.99(100)** 273(49) 246(25) 245(31) 230(63) 229(69) 201(53) 175(33) **169(48)** 166(44) 145(25) 123(60)		
			-	715.0575	[M_2_-2H]^2−^	C_54_H_46_O_46_	−8.8	20	715	**706(100)** 705(14) 697(83) 691(37) 651(15) 631(20) 613(26) 601(15) **301(14)** 291(14) **169(38)**	Ellagitannin *m*/*z* 1431	
**L16**	5.59	222 274	-	1431.1285	[M_2_-H]^−^	C_54_H_47_O_46_	−4.1	40	715	403(32) **300.99(100)** 298(41) 286(42) 275(40) 273(67) 260(45) 245(45) 229(93) 219(34) 217(36) 214(45) 191(38) 189(47) **169(42)** 123(62)	Ellagitannin *m*/*z* 1431	
**L17**	5.70	224 265	-	705.0447	[M_1_-2H]^2−^	C_54_H_42_O_45_	−8.2	40	705	**300.99(100)** 287(47) 275(46) 229(63) 217(47) 214(50) 167(67) 123(48)	Ellagitannin *m*/*z* 1411	
			-	1411.0998	[M_1_-H]^−^	C_54_H_43_O_45_	−5.9					
			-	714.0498	[M_2_-2H]^2−^	C_54_H_44_O_46_	−8.3	10	714	**705(100)** 702(12) 696(24) 691(52) 631(9) 613(22) 601(10) 599(12) **300.99(9)** 299(11) 169(10)	Ellagitannin *m*/*z* 1429	
			-	1429.1111	[M_2_-H]^−^	C_54_H_45_O_46_	−5.4	30	714	613(57) 601(34) 431(31) 401(30) **300.99(81)** 299(43) 275(37) 273(86) **261(100)** 247(33) 229(48) 169(55) 167(65)		
**L19**	6.03	224 256 274 350sh	-	631.0575	[M-H]^−^	C_27_H_19_O_18_	−0.3	30	631	613(55) 465(30) 464(24) 461(23) **445(100)** 443(39) **300.99(58)** 298(48) 272(48) 166(64) 123(36) 102(21)	Ellagitannin *m*/*z* 631(similar to castalin/vescalin)	[37]
			-	653.0390	[M+Na-2H]^−^	C_27_H_18_O_18_Na	−1.0	30	653	635(33) 625(23) 461(23) 433(34) 322(36) **300.99(100)** 299(60) 271(31) 169(14)		
**L20**	6.25	222 278 330sh	-	635.0882	[M-H]^−^	C_27_H_23_O_18_	−1.2	30	635	635(52) 483(37) 465(99) 423(11) 313(68) 295(15) **169(100)** 125(27)	Tri-*O*-galloyl-hexose	[38,55]
**L25**	7.13	224 255 370sh	-	779.0381	[M-H]^−^	C_34_H_19_O_22_	1.0	20	779	779(11) 761(11) **751(100)** 733(37) 449(23) **300.99(84)** 299(57) 286(12) 275(13) 273(16) 261(12) 242.9(9) 214.9(9)	Ellagitannin *m*/*z* 779	
			-	797.0486	[M-H]^−^	C_34_H_21_O_23_	0.9	20	797	797(13) 779(22) 773(10) 753(36) **751(100)** 735(25) 733(39) 725(11) 449(24) 316(14) **300.99(90)** 299(90) 291(35) 288(11) 286(11) 275(28) 273(73) 270(20) 247(23)	Ellagitannin *m*/*z* 797	
**L25**	7.13	224 255 370sh	-	815.0578	[M-H]^−^	C_34_H_23_O_24_	−0.8	20	815	815(13) 797(27) 779(40) 753(71) **751(100)** 735(51) 733(55) 725(18) 709(11) 707(14) 449(18) 435(14) 316(12) **300.99(91)** 299(57) 291(45) 275(24) 273(55) 270(14) 261(12) 247(61) 245(20)	Ellagitannin *m*/*z* 815	
**L27**	7.33	252 292sh 302 354sh 368	-	300.9991	[M-H]^−^	C_14_H_5_O_8_	0.4	40	301	299(36) 283(57) 229(34) 228(28) 217(35) 201(53) 200(51) 185(33) 173(58) 172(34) 161(38) 157(37) **145(100)** 133(31) 129(26) 117(50)	Ellagic acid *	[40,55]
**L28**	7.69	212 222 276	-	791.0631	[M-2H]^2−^			30	791	787(18) 785(26) 781(19) 767(17) **765(100)** 753(28) 721(14) 613(15) 601(43) 598(14) **300.99(53)** 299(30) 273(22) 249(21) 211(18) **169(20)**	Ellagitannin *m*/*z* 791	
**L29**	7.80	224 256 265sh 295sh 353	-	477.0669	[M-H]^−^	C_21_H_17_O_13_	−1.2	20	477	**301(100)** 179(5) 151(5)	Quercetin-3-*O*-glucuronide	[33,40]
			+	479.0798	[M+H]^+^	C_21_H_19_O_13_	−4.6	20	479	**303(100)** 159(2) 141(1) 113(5)		
			+	501.0624	[M+Na]^+^	C_21_H_18_O_13_Na	−3.1	20	501	**325(100)** 199(7) 140(3)		
**L30**	7.94	228 290 335sh	-	449.1085	[M-H]^−^	C_21_H_21_O_11_	−1.0	20	449	323(4) 303(14) 285(67) 241(4) 179(14) **151(100)** 125(16) 107(8)	Astilbin (taxifolin-3-*O*-rhamnoside)	[33,41,42]
			+	473.1041	[M+Na]^+^	C_21_H_22_O_11_Na	−2.8					

* annotation confirmed by comparison to authentic standard analysis.

## Supplementary Materials

Supplementary materials can be found at https://www.mdpi.com/1422-0067/20/22/5775/s1.

## References

[1] Harborne J.B., Williams C.A. (2000). Advances in flavonoid research since 1992. Phytochemistry.

[2] Bravo L. (1998). Polyphenols: Chemistry, dietary sources, metabolism, and nutritional significance. Nut Rev..

[3] Mellway R.D., Constabel C.P. (2009). Metabolic engineering and potential functions of proanthocyanidins in poplar. Plant. Signal. Behav..

[4] Latouche G., Bellow S., Poutaraud A., Meyer S., Cerovic Z.G. (2013). Influence of constitutive phenolic compounds on the response of grapevine (*Vitis vinifera* L.) leaves to infection by *Plasmopara Viticola*. Planta.

[5] Caretto S., Linsalata V., Colella G., Mita G., Lattanzio V. (2015). Carbon Fluxes between Primary Metabolism and Phenolic Pathway in Plant Tissues under Stress. Int. J. Mol. Sci..

[6] Barbehenn R.V., Peter Constabel C. (2011). Tannins in plant-herbivore interactions. Phytochemistry.

[7] Cheynier V., Comte G., Davies K.M., Lattanzio V., Martens S. (2013). Plant phenolics: Recent advances on their biosynthesis, genetics, and ecophysiology. Plant. Physiol. Biochem..

[8] Mandal S.M., Chakraborty D., Dey S. (2010). Phenolic acids act as signaling molecules in plant-microbe symbioses. Plant. Signal. Behav..

[9] Teixeira A., Eiras-Dias J., Castellarin S.D., Geros H. (2013). Berry phenolics of grapevine under challenging environments. Int. J. Mol. Sci..

[10] Krol A., Amarowicz R., Weidner S. (2015). The effects of cold stress on the phenolic compounds and antioxidant capacity of grapevine (*Vitis vinifera* L.) leaves. J. Plant. Physiol..

[11] Magnin-Robert M., Spagnolo A., Boulanger A., Joyeux C., Clément C., Abou-Mansour E., Fontaine F. (2016). Changes in Plant Metabolism and Accumulation of Fungal Metabolites in Response to Esca Proper and Apoplexy Expression in the Whole Grapevine. Phytopathology.

[12] Garcia-Seco D., Zhang Y., Gutierrez-Manero F.J., Martin C., Ramos-Solano B. (2015). Application of *Pseudomonas fluorescens* to Blackberry under Field Conditions Improves Fruit Quality by Modifying Flavonoid Metabolism. PLoS ONE.

[13] Singh U.P., Sarma B.K., Singh D.P. (2003). Effect of plant growth-promoting rhizobacteria and culture filtrate of *Sclerotium rolfsii* on phenolic and salicylic acid contents in chickpea (*Cicer arietinum*). Curr. Microbiol..

[14] Lavania M., Chauhan P.S., Chauhan S.V., Singh H.B., Nautiyal C.S. (2006). Induction of plant defense enzymes and phenolics by treatment with plant growth-promoting rhizobacteria *Serratia marcescens* NBRI1213. Curr. Microbiol..

[15] Ait Barka E., Nowak J., Clément C. (2006). Enhancement of chilling resistance of inoculated grapevine plantlets with a plant growth-promoting rhizobacterium, *Burkholderia phytofirmans* strain PsJN. Appl. Environ. Microbiol..

[16] Portu J., Gonzalez-Arenzana L., Hermosin-Gutierrez I., Santamaria P., Garde-Cerdan T. (2015). Phenylalanine and urea foliar applications to grapevine: Effect on wine phenolic content. Food Chem..

[17] Singh A., Jain A., Sarma B.K., Upadhyay R.S., Singh H.B. (2014). Rhizosphere competent microbial consortium mediates rapid changes in phenolic profiles in chickpea during *Sclerotium rolfsii* infection. Microbiol. Res..

[18] Vejan P., Abdullah R., Khadiran T., Ismail S., Nasrulhaq Boyce A. (2016). Role of Plant Growth Promoting Rhizobacteria in Agricultural Sustainability—A Review. Molecules.

[19] Martin C., Zhang Y., Tonelli C., Petroni K. (2013). Plants, diet, and health. Annu. Rev. Plant. Biol..

[20] Girish N., Umesha S. (2007). Effect of plant growth promoting rhizobacteria on bacterial canker of tomato. Arch. Phytopathol. Plant. Prot..

[21] Gutierrez Mañero F.J., Ramos B., Lucas J.A., Probanza A., Barrientos M.L. (2003). Systemic induction of terpenic compounds in *D. Lanata*. J. Plant. Physiol..

[22] Zhang S., Reddy M.S., Kloepper J.W. (2004). Tobacco growth enhance-ment and blue mold protection by rhizobacteria: Relationship between plant growth promotion and systemic disease protection by PGPR strain. Plant. Soil.

[23] Theocharis A., Bordiec S., Fernandez O., Paquis S., Dhondt-Cordelier S., Baillieul F., Clément C., Barka E.A. (2012). *Burkholderia phytofirmans* PsJN primes *Vitis vinifera* L. and confers a better tolerance to low nonfreezing temperatures. Mol. Plant. Microbe.

[24] Fernandez O., Theocharis A., Bordiec S., Feil R., Jacquens L., Clément C., Fontaine F., Barka E.A. (2012). *Burkholderia phytofirmans* PsJN acclimates grapevine to cold by modulating carbohydrate metabolism. Mol. Plant. Microbe.

[25] Miotto-Vilanova L., Jacquard C., Courteaux B., Wortham L., Michel J., Clément C., Ait Barka E., Sanchez L. (2016). *Burkholderia phytofirmans* PsJN confers grapevine resistance against *Botrytis cinerea* via a direct antimicrobial effect combined with a better resource mobilization. Front. Plant. Sci..

[26] Issa A., Esmaeel Q., Sanchez L., Courteaux B., Gibon Y., Ballias P., Clément C., Jacquard C., Vaillant-Gaveau N., Aït Barka E. (2018). Impact of *Paraburkholderia phytofirmans* strain PsJN on tomato (*Lycopersicon esculentum* L.) under high temperature. Front. Plant. Sci..

[27] Su F., Gilard F., Guérard F., Citerne S., Clément C., Vaillant-Gaveau N., Dhondt-Cordelier S. (2016). Spatio-temporal Responses of *Arabidopsis* Leaves in Photosynthetic Performance and Metabolite Contents to *Burkholderia phytofirmans* PsJN. Front. Plant. Sci..

[28] Fernandez O., Vandesteene L., Feil R., Baillieul F., Lunn J.E., Clément C. (2012). Trehalose metabolism is activated upon chilling in grapevine and might participate in *Burkholderia phytofirmans* induced chilling tolerance. Planta.

[29] Valette M., Rey M., Gerin F., Comte G., Wisniewski-Dye F. (2019). A common metabolomic signature is observed upon inoculation of rice roots with various rhizobacteria. J. Integr. Plant. Biol..

[30] Vannozzi A., Dry I.B., Fasoli M., Zenoni S., Lucchin M. (2012). Genome-wide analysis of the grapevine stilbene synthase multigenic family: Genomic organization and expression profiles upon biotic and abiotic stresses. BMC Plant Biol..

[31] Nuengchamnong N., Ingkaninan K. (2009). On-line characterization of phenolic antioxidants in fruit wines from family myrtaceae by liquid chromatography combined with electrospray ionization tandem mass spectrometry and radical scavenging detection. Food Sci. Technol..

[32] Silva F.L.D.N., Schmidt E.M., Messias C.L., Eberlin M.N. (2014). Quantitation of organic acids in wine and grapes by direct infusion electrospray ionization mass spectrometry. Anal. Methods UK.

[33] Souquet J.M., Labarbe B., Le Guerneve C., Cheynier V., Moutounet M. (2000). Phenolic composition of grape stems. J. Agric. Food Chem..

[34] Flamini R. (2013). Recent applications of mass spectrometry in the study of grape and wine polyphenols. ISRN Spectrosc..

[35] Khoza B.S., Gbashi S., Steenkamp P.A., Njobeh P.B., Madala N.E. (2016). Identification of hydroxylcinnamoyl tartaric acid esters in Bidens pilosa by UPLC-tandem mass spectrometry. S. Afr. J. Bot..

[36] Fang N., Yu S., Prior R.L. (2002). LC/MS/MS characterization of phenolic constituents in dried plums. J. Agric. Food Chem..

[37] Sanz M., de Simon B.F., Cadahia E., Esteruelas E., Munoz A.M., Hernandez T., Estrella I., Pinto E. (2012). LC-DAD/ESI-MS/MS study of phenolic compounds in ash (*Fraxinus excelsior* L. and *F. americana* L.) heartwood. Effect of toasting intensity at cooperage. J. Mass Spectrom..

[38] Abu-Reidah I.M., Ali-Shtayeh M.S., Jamous R.M., Arraez-Roman D., Segura-Carretero A. (2015). HPLC-DAD-ESI-MS/MS screening of bioactive components from *Rhus coriaria* L. (Sumac) fruits. Food Chem..

[39] Mena P., Calani L., Dall’Asta C., Galaverna G., Garcia-Viguera C., Bruni R., Crozier A., Del Rio D. (2012). Rapid and comprehensive evaluation of (poly)phenolic compounds in pomegranate (*Punica granatum* L.) juice by UHPLC-MSn. Molecules.

[40] Aaby K., Ekeberg D., Skrede G. (2007). Characterization of phenolic compounds in strawberry (Fragaria x ananassa) fruits by different HPLC detectors and contribution of individual compounds to total antioxidant capacity. J. Agric. Food Chem..

[41] Zhao M., Xu J., Qian D., Guo J., Jiang S., Shang E.X., Duan J.A. (2014). Identification of astilbin metabolites produced by human intestinal bacteria using UPLC-Q-TOF/MS. Biomed. Chromatogr..

[42] Billet K., Houille B., Duge de Bernonville T., Besseau S., Oudin A., Courdavault V., Delanoue G., Guerin L., Clastre M., Giglioli-Guivarc’h N. (2018). Field-Based Metabolomics of *Vitis vinifera* L. Stems Provides New Insights for Genotype Discrimination and Polyphenol Metabolism Structuring. Front. Plant. Sci..

[43] Li X., Zhang Y., Zeng X., Yang L., Deng Y. (2011). Chemical profiling of bioactive constituents in *Sarcandra glabra* and its preparations using ultra-high-pressure liquid chromatography coupled with LTQ Orbitrap mass spectrometry. Rapid Commun. Mass Spectrom..

[44] Cantwell M.I., Peiser G., Mercado-Silva E. (2002). Induction of chilling injury in jicama (*Pachyrhizus erosus*) roots: Changes in texture, color and phenolics. Postharvest Biol. Technol..

[45] Rockenbach I.I., Jungfer E., Ritter C., Santiago-Schübel B., Thiele B., Fett R., Galenza R. (2012). Characterization of flavan-3-ols in seeds of grape pomace by CE, HPLC-DAD-MSn and LC-ESI-FTICR-MS. Food Res. Int..

[46] Sandhu A.K., Gu L. (2010). Antioxidant capacity, phenolic content, and profiling of phenolic compounds in the seeds, skin, and pulp of *Vitis rotundifolia* (Muscadine Grapes) As determined by HPLC-DAD-ESI-MS(n). J. Agric. Food Chem..

[47] Montero L., Herrero M., Prodanov M., Ibanez E., Cifuentes A. (2013). Characterization of grape seed procyanidins by comprehensive two-dimensional hydrophilic interaction x reversed phase liquid chromatography coupled to diode array detection and tandem mass spectrometry. Anal. Bioanal. Chem..

[48] Nawrot-Hadzik I., Slusarczyk S., Granica S., Hadzik J., Matkowski A. (2019). Phytochemical Diversity in Rhizomes of Three Reynoutria Species and their Antioxidant Activity Correlations Elucidated by LC-ESI-MS/MS Analysis. Molecules.

[49] Bavaresco L., Fregoni M., Trevisan M., Mattivi F., Vrhovsek U., Falchetti R. (2002). The occurrence of the stilbene piceatannol in grapes. Vitis.

[50] Rodriguez-Cabo T., Rodriguez I., Lopez P., Ramil M., Cela R. (2014). Investigation of liquid chromatography quadrupole time-of-flight mass spectrometry performance for identification and determination of hydroxylated stilbene antioxidants in wine. J. Chromatogr..

[51] Flamini R., Zanzotto A., de Rosso M., Lucchetta G., Vedova A.D., Bavaresco L. (2016). Stilbene oligomer phytoalexins in grape as a response to *Aspergillus carbonarius* infection. Physiol. Mol. Plant. Pathol..

[52] Tavares I.M.C., Silva Lago-Vanzela E., Portugal Gomes Rebello L., Mota Ramos A., Gómez-Alonso S., García-Romero E., Da-Silva R., Hermosín-Gutiérrez I. (2016). Comprehensive study of the phenolic composition of the edible parts of jambolan fruit (*Syzygium cumini* (L.) Skeels). Food Res. Int..

[53] Romani A., Campo M., Pinelli P. (2012). HPLC/DAD/ESI-MS analyses and anti-radical activity of hydrolyzable tannins from different vegetal species. Food Chem..

[54] Dorta E., González M., Lobo M.G., Sánchez-Moreno C., de Ancos B. (2014). Screening of phenolic compounds in by-product extracts from mangoes (*Mangifera indica* L.) by HPLC-ESI-QTOF-MS and multivariate analysis for use as a food ingredient. Food Res. Int..

[55] Gu D., Yang Y., Bakri M., Chen Q., Xin X., Aisa H.A. (2013). A LC/QTOF-MS/MS application to investigate chemical compositions in a fraction with protein tyrosine phosphatase 1B inhibitory activity from *Rosa rugosa* flowers. Phytochem. Anal..

[56] Chamam A., Sanguin H., Bellvert F., Meiffren G., Comte G., Wisniewski-Dye F., Bertrand C., Prigent-Combaret C. (2013). Plant secondary metabolite profiling evidences strain-dependent effect in the *Azospirillum-Oryza sativa* association. Phytochemistry.

[57] Gupta R., Tiwari S., Saikia S.K., Shukla V., Singh R., Singh S.P., Kumar P.V., Pandey R. (2015). Exploitation of microbes for enhancing bacoside content and reduction of *Meloidogyne incognita* infestation in *bacopa monnieri* L.. Protoplasma.

[58] Le Roy J., Huss B., Creach A., Hawkins S., Neutelings G. (2016). Glycosylation Is a Major Regulator of Phenylpropanoid Availability and Biological Activity in Plants. Front. Plant. Sci..

[59] Sanchez-Maldonado A.F., Schieber A., Ganzle M.G. (2011). Structure-function relationships of the antibacterial activity of phenolic acids and their metabolism by lactic acid bacteria. J. Appl. Microbiol..

[60] Bernal-Mercado A.T., Vazquez-Armenta F.J., Tapia-Rodriguez M.R., Islas-Osuna M.A., Mata-Haro V., Gonzalez-Aguilar G.A., Lopez-Zavala A.A., Ayala-Zavala J.F. (2018). Comparison of Single and Combined Use of Catechin, Protocatechuic, and Vanillic Acids as Antioxidant and Antibacterial Agents against Uropathogenic *Escherichia Coli* at Planktonic and Biofilm Levels. Molecules.

[61] Landete J.M. (2011). Ellagitannins, ellagic acid and their derived metabolites: A review about source, metabolism, functions and health. Food Res. Int..

[62] Bézier A., Lambert B., Baillieul F. (2002). Study of defense-related gene expression in grapevine leaves and berries Infected with *Botrytis cinerea*. Eur. J. Plant. Pathol..

[63] Mutawila C., Stander C., Halleen F., Vivier M.A., Mostert L. (2017). Response of *Vitis vinifera* cell cultures to *Eutypa lata* and *Trichoderma atroviride* culture filtrates: Expression of defence-related genes and phenotypes. Protoplasma.

[64] Dufour M.C., Magnin N., Dumas B., Vergnes S., Corio-Costet M.F. (2016). High-throughput gene-expression quantification of grapevine defense responses in the field using microfluidic dynamic arrays. BMC Genom..

[65] Nopo-Olazabal C., Condori J., Nopo-Olazabal L., Medina-Bolivar F. (2014). Differential induction of antioxidant stilbenoids in hairy roots of *Vitis rotundifolia* treated with methyl jasmonate and hydrogen peroxide. Plant Physiol. Biochem..

[66] Kortekamp A. (2006). Expression analysis of defence-related genes in grapevine leaves after inoculation with a host and a non-host pathogen. Plant Physiol. Biochem..

[67] Akinwumi B.C., Bordun K.M., Anderson H.D. (2018). Biological Activities of Stilbenoids. Int. J. Mol. Sci..

[68] Murias M., Jager W., Handler N., Erker T., Horvath Z., Szekeres T., Nohl H., Gille L. (2005). Antioxidant, prooxidant and cytotoxic activity of hydroxylated resveratrol analogues: Structure-activity relationship. Biochem. Pharm..

[69] Ovesna Z., Kozics K., Bader Y., Saiko P., Handler N., Erker T., Szekeres T. (2006). Antioxidant activity of resveratrol, piceatannol and 3,3′,4,4′,5,5′-hexahydroxy-trans-stilbene in three leukemia cell lines. Oncol. Rep..

[70] Temsamani H., Krisa S., Decossas-Mendoza M., Lambert O., Merillon J.M., Richard T. (2016). Piceatannol and Other Wine Stilbenes: A Pool of Inhibitors against alpha-Synuclein Aggregation and Cytotoxicity. Nutrients.

[71] Cantos E., Espin J.C., Tomas-Barbera F.A. (2002). Varietal differences among the polyphenol profiles of seven table grape cultivars studied by LC-DAD-MS-MS. J. Agric. Food Chem..

[72] Xia E.Q., Deng G.F., Guo Y.J., Li H.B. (2010). Biological activities of polyphenols from grapes. Int. J. Mol. Sci..

[73] Armero J., Requejo R., Jorrin J., Lopez-Valbuena R., Tena M. (2001). Release of phytoalexins and related iso-flavonoids from intact chickpea seedlings elicited withreduced glutathione at root level. Plant Physiol. Biochem..

[74] Weston L.A., Mathesius U. (2013). Flavonoids: Their structure, biosynthesis and role in the rhizosphere, including allelopathy. J. Chem. Ecol..

[75] Hassan S., Mathesius U. (2012). The role of flavonoids in root-rhizosphere signalling: Opportunities and challenges for improving plant-microbe interactions. J. Exp. Bot..

[76] Yilmaz Y., Toledo R.T. (2004). Major flavonoids in grape seeds and skins: Antioxidant capacity of catechin, epicatechin, and gallic acid. J. Agric. Food Chem..

[77] Ursini F., Rapuzzi I., Toniolo R., Tubaro F., Bontempelli G. (2001). Characterization of antioxidant effect of procyanidins. Methods Enzym..

[78] Larrosa M., Garcia-Conesa M.T., Espin J.C., Tomas-Barberan F.A. (2010). Ellagitannins, ellagic acid and vascular health. Mol. Asp. Med..

[79] Braicu C., Pilecki V., Balacescu O., Irimie A., Neagoe I.B. (2011). The relationships between biological activities and structure of flavan-3-ols. Int. J. Mol. Sci..

[80] Doshi P., Adsule P., Banerjee K. (2006). Phenolic composition and antioxidant activity in grapevine parts and berries (*Vitis vinifera* L.) cv. Kishmish Chornyi (Sharad Seedless) during maturation. Int. J. Food Sci..

[81] Dietrich H., Pour-Nikfardjam M.S., König H., Unden G., Fröhlich J. (2009). Influence of Phenolic Compounds and Tannins on Wine-Related Microorganisms. Biology of Microorganisms on Grapes, in Must and in Wine.

[82] Irani N.G., Grotewold E. (2005). Light-induced morphological alteration in anthocyanin-accumulating vacuoles of maize cells. BMC Plant Biol..

[83] Fontes N., Geros H., Delrot S. (2011). Grape Berry Vacuole: A Complex and Heterogeneous Membrane System Specialized in the Accumulation of Solutes. Am. J. Enol. Vitic..

[84] Shih C.H., Siu S.O., Ng R., Wong E., Chiu L.C., Chu I.K., Lo C. (2007). Quantitative analysis of anticancer 3-deoxyanthocyanidins in infected sorghum seedlings. J. Agric. Food Chem..

[85] Shih P.H., Yeh C.T., Yen G.C. (2007). Anthocyanins induce the activation of phase II enzymes through the antioxidant response element pathway against oxidative stress-induced apoptosis. J. Agric. Food Chem..

[86] Bassolino L., Zhang Y., Schoonbeek H.J., Kiferle C., Perata P., Martin C. (2013). Accumulation of anthocyanins in tomato skin extends shelf life. New Phytol..

[87] Zhang Y., Butelli E., De Stefano R., Schoonbeek H.J., Magusin A., Pagliarani C., Wellner N., Hill L., Orzaez D., Granell A. (2013). Anthocyanins double the shelf life of tomatoes by delaying overripening and reducing susceptibility to gray mold. Curr. Biol..

[88] Goetz G., Fkyerat A., Metais N., Kunz M., Tabacchi R., Pezet R., Pont V. (1999). Resistance factors to grey mould in grape berries: Identification of some phenolics inhibitors of *Botrytis cinerea* stilbene oxidase. Phytochemistry.

[89] King E.O., Ward M.K., Raney D.E. (1954). Two simple media for the demonstration of pyocyanin and fluorescin. J. Lab. Clin. Med..

