# Supplementary material for: Impact of Paraburkholderia phytofirmans PsJN on Grapevine Phenolic Metabolism
Source: Int J Mol Sci. 2019 Nov 16;20(22):5775. doi: 10.3390/ijms20225775 (PMC6888286; doi:10.3390/ijms20225775)
Supplement: Supplementary file 1 [file ijms-20-05775-s001.pdf]

Supplemental data

Impact of *Paraburkholderia phytofirmans* PsJN on grapevine phenolics metabolism

Lidiane Miotto-Vilanova[1#], Barbara Courteaux[1#], Rosa Padilla[2], Fanja Rabenoelina[1], Cédric Jacquard[1], Christophe Clément[1], Gilles Comte[2], Céline Lavire[2], Essaïd Ait Barka[1], Isabelle Kerzaon[2] and Lisa Sanchez[1*]

[1] Unité de Recherche EA 4707 Résistance Induite et Bioprotection des Plantes (RIBP), SFR Condorcet FR CNRS 3417, Université de Reims Champagne-Ardenne, Reims, France ; lidianemiotto@gmail.com (L.M.-V.); barbara.courteaux@univ-reims.fr (B.C.); clarisse.rabenoelina@univ-reims.fr (F.R.) ; cedric.jacquard@univ-reims.fr (C.J.) ; christophe.clement@univ-reims.fr (C.C.) ; ea.barka@univ-reims.fr (E.A.B.)
[2] Ecologie Microbienne, CNRS, INRA, UMR 5557, Université Lyon 1, Villeurbanne, France, gilles.comte@univ-lyon1.fr (G.C.) ; celine.lavire@univ-lyon1.fr (C.L.) ; isabelle.kerzaon@univ-lyon1.fr (I.K.)
# These authors contributed equally to this paper
* Correspondence: lisa.sanchez@univ-reims.fr; Tel.: +33-326-913-436

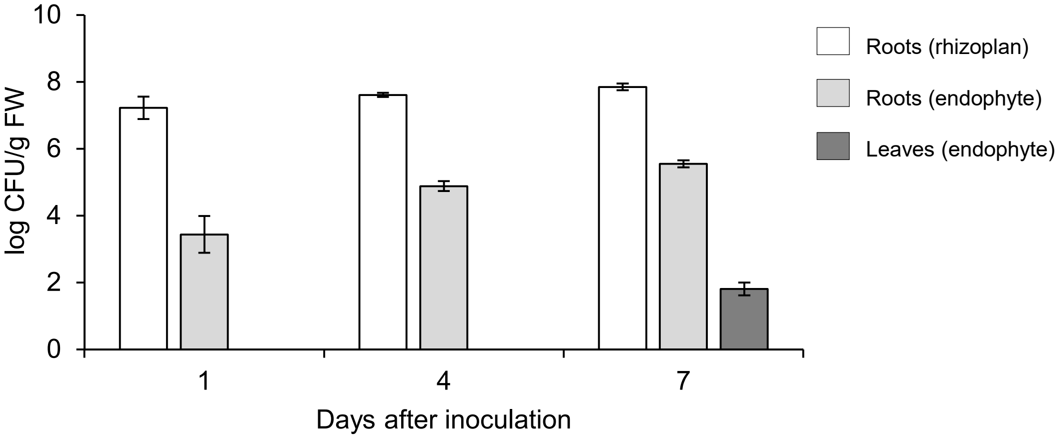

Supplemental figure S1: Grapevine colonization by *P. phytofirmans* PsJN. Values shown are means +/- SD of three independent repetitions (each repetition was realized in triplicates)

(a)

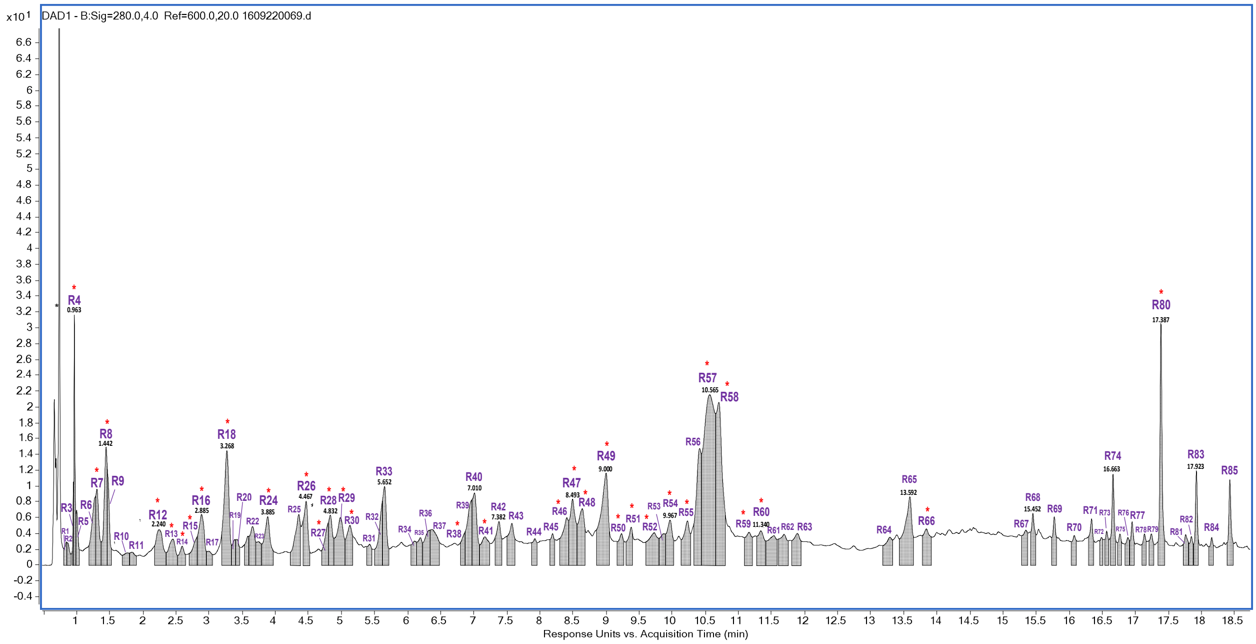

(b)

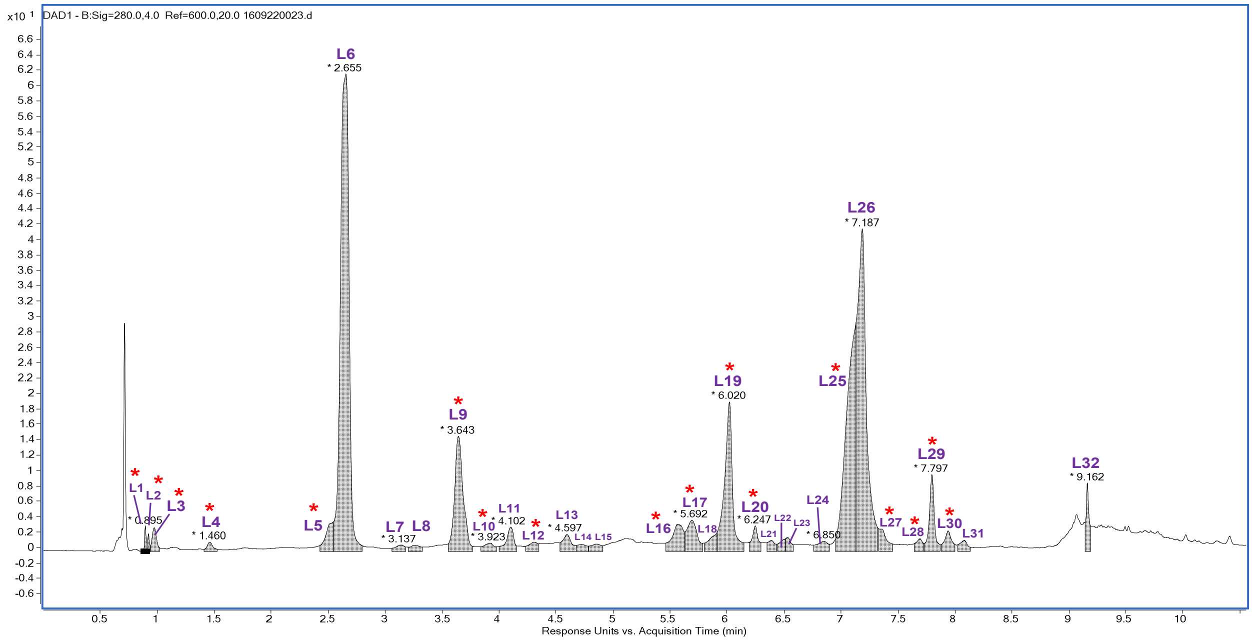

Supplemental figure S2: Analyses by UHPLC-UV/DAD-MS QTOF of phenolic compounds extracted from grapevine roots (a) and leaves (b) inoculated or not with *P. phytofirmans* strain PsJN. The chromatogram at 280 nm presented corresponds to the analysis of the QC (Quality control) sample for (a) roots and (b) leaves of the first biological repeat. The integrated and numbered chromatographic peaks were taken into account for the comparison of the profiles between the two conditions (control and bacterized). * indicates the peak wtih a significant difference (P <0.05, Student t-test) between the two conditions.

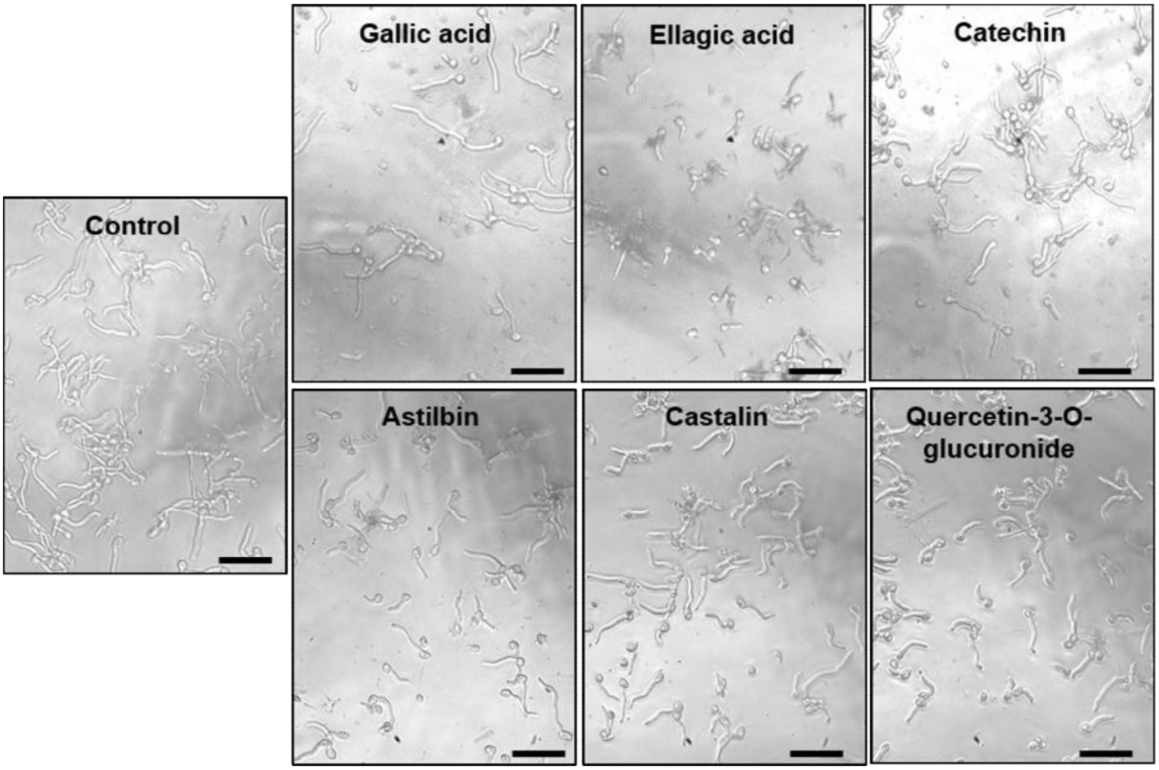

Supplemental figure S3: Effect of molecules on *Botrytis cinerea* spore germination. Conidia were placed in growth medium supplemented with solutions of molecules at 0.1mg/mL. Germ tubes were observed by inverted light microscopy 8h later. Scale: 50 µm.

Supplemental Table S1: Primers used in this study

Gene symbol	Gene name	Forward primer sequence	Reverse primer sequence	Reference
PAL	phenylalanine-ammonia lyase	TCCTCCCGGAAAACAGCTG	TCCTCCAAATGCCTCAAATCA	Varnier et al. 2009
STS	stilbene synthase	AGGAAGCAGCATTGAAGGCTC	TGCACCAGGCATTTCTACACC	Varnier et al. 2010
CHS1	chalcone synthase	CGAAGGAGCAATCGACGGA	GTCGCTGATGCCTATCGGAG	this study
CHS2	chalcone synthase	GGAAGATGGGAATGGCTGCT	GAGAGAAGGCACAGGGACAC	this study
CHS3	chalcone synthase	GCCCTAAAGCCCGAGAAGTT	AGCCGACTTCCTCCTCATCT	this study
CHI1	chalcone isomerase 1	GCAGAAGCCAAAGCCATTGA	GCCGATGATGGACTCCAGTAC	this study
CHI2	chalcone isomerase 2	TCCAGATCAAGTTCACAGCA	GAAACAAGAGCCTCAAAGAA	Gutha et al. 2010
FLS1	flavonol synthase	CAGGGCTTGCAGGTTTTTAG	GGGTCTTCTCCTTGTTCACG	Gutha et al. 2010
LAR1	leucoanthocyanidin reductase 1	AAATGAACTCGCATCTGTGT	CTGTGGGATGATGTTTTCTC	Gutha et al. 2010
LAR2	leucoanthocyanidin reductase 2	TGATATCAGCTGTGGGTGGA	CCCAAATTCTGATGGAAGGA	Gutha et al. 2010
LDOX	leucoanthocyanidin dioxygenase	ATGAGGGCAAGTGGGTGACA	TTGACCAGTCCCCTGTGAAGA	this study
ANR	anthocyanidin reductase	GCTGCTGTTACCATCAATCA	GCAGGATAGCCCCAAGTAGG	Gutha et al. 2010
UFGT	UDP-glucose:flavonoid 3-Oglucosyltransferase	GGGATGGTAATGGCTGTGG	ACATGGGTGGAGAGTGAGTT	Gutha et al. 2010

- Gutha, L.R.; Casassa, L.F.; Harbertson, J.F.; Naidu, R.A. Modulation of flavonoid biosynthetic pathway genes and anthocyanins due to virus infection in grapevine (*Vitis vinifera* L.) leaves. BMC Plant Biol **2010**, *10*, 187. doi: 10.1186/1471-2229-10-187.
- Varnier, A.L.; Sanchez, L.; Vatsa, P.; Boudesocque, L.; Garcia-Brugger, A.; Rabenoelina, F.; Sorokin, A.; Renault, J.H.; Kauffmann S.; Pugin, A.; Clément, C.; Baillieul, F.; Dorey, S. Bacterial rhamnolipids are novel MAMPs conferring resistance to *Botrytis cinerea* in grapevine. Plant Cell Environ **2009**, *32*, 178-193. doi: 10.1111/j.1365-3040.2008.01911.x.
